# DispatchMAS: fusing taxonomy and artificial intelligence agents for emergency medical services

**DOI:** 10.1186/s12873-026-01540-9

**Published:** 2026-03-18

**Authors:** Xiang Li, Huizi Yu, Wenkong Wang, Yiran Wu, Jiayan Zhou, Wenyue Hua, Xinxin Lin, Wenjia Tan, Lexuan Zhu, Bingyi Chen, Guang Chen, Ming-Li Chen, Yang Zhou, Zhao Li, Themistocles L. Assimes, Yongfeng Zhang, Qingyun Wu, Xin Ma, Lingyao Li, Lizhou Fan

**Affiliations:** 1 Shandong University, Jinan, Shandong China; 2 The Chinese University of Hong Kong, Sha Tin, NT Hong Kong SAR, China; 3 Pennsylvania State University, University Park, PA USA; 4 Stanford University School of Medicine, Stanford, CA USA; 5 University of California, Santa Barbara, CA USA; 6 University of Macau, Macau SAR, China; 7 New York University, New York, NY USA; 8 Broad Institute of MIT and Harvard, Cambridge, MA USA; 9 The University of Hong Kong, Hong Kong SAR, China; 10 Chinese Academy of Medical Sciences and Peking Union Medical College, Beijing, China; 11 Chinese Academy of Medical Sciences, Beijing, China; 12 Rutgers University, New Brunswick, NJ USA; 13 University of South Florida, Tampa, FL USA

**Keywords:** Emergency Medical Services, Multi-Agent Systems, Taxonomy, Fact Commons, Prehospital Care

## Abstract

**Background:**

Emergency medical dispatch is a critical, high-stakes process where dispatcher decisions directly impact patient outcomes. While standardized protocols exist, they are challenged by factors like caller distress, ambiguous symptom descriptions, and high cognitive load. The convergence of Large Language Models (LLMs) and Multi-Agent Systems (MAS) offers a novel opportunity to augment human dispatchers. This study aimed to develop and evaluate a taxonomy-grounded, LLM-powered multi-agent system for simulating realistic clinician’s medical dispatch scenarios.

**Methods:**

We first constructed a clinically curated taxonomy and fact commons for emergency dispatch, defining 32 Chief Complaints based on national standards, six distinct caller identities derived from real-world electronic health records (Medical Information Mart for Intensive Care III [MIMIC-III]), and a standardized six-phase call protocol. Using this framework, we developed a multi-agent simulation system featuring a Caller Agent and a Dispatcher Agent. The system, built on the AutoGen multi-agent framework for large language models (AutoGen), grounds agent interactions in the fact commons to ensure clinical plausibility and mitigate misinformation. We designed a hybrid evaluation combining expert clinical assessment, automated linguistic analysis, and operational performance dynamics auditing. Four physicians evaluated 100 simulated dispatch cases for “Guidance Efficacy” and “Dispatch Effectiveness” using a structured questionnaire. Automated metrics assessed sentiment, emotion, readability, and politeness of agent-generated dialogue. Operational performance dynamics analyses showed phase-dependent efficiency peaking during Assessment and faster pacing in life-critical events.

**Results:**

Human evaluation, with substantial inter-rater agreement (Gwet’s AC1 $> \,\mathrm{0.70}$, confirmed the system’s high performance. It demonstrated excellent Dispatch Effectiveness (e.g., 94% contacting the correct potential other agents) and Guidance Efficacy (advice provided in 91% of cases), both rated highly by physicians. Algorithmic metrics corroborated these findings, indicating a predominantly neutral affective profile (73.7% neutral sentiment; 90.4% neutral emotion), high readability (Flesch 80.9), and a consistently polite style (60.0% polite; 0% impolite). Operational performance evaluation further showed urgency-adaptive pacing: for life-critical events, information completeness rose faster and plateaued earlier while converging to comparable end-of-call completeness across complaint types. The agent also responded more rapidly in life-critical scenarios (1.8 s per dispatcher turn vs 2.1–2.4 s), indicating accelerated early questioning without loss of overall coverage.

**Conclusion:**

Our LLM-based MAS simulates diverse, clinically plausible dispatch scenarios with high fidelity. The resulting platform provides a controlled environment for analyzing dispatcher–caller interactions, stress-testing protocol variants, and deriving structured design patterns that may inform future real-time decision support. Our simulation-based tools could serve as an intermediate step between offline method development and eventual integration into emergency response workflows.

**Supplementary information:**

The online version contains supplementary material available at 10.1186/s12873-026-01540-9.

## Background

Emergency medical dispatch, where critical triage decisions are made based on limited caller information, is a pivotal step in prehospital care that directly impacts patient morbidity and mortality. National standards such as the Emergency Medical Dispatch Priority Reference System (EMDPRS) [1, 2] have been developed to promote consistency and safety across dispatch operations by guiding call-takers through structured, symptom-based protocols [3]. These systems aim to ensure timely and accurate identification of emergencies and the provision of life-saving pre-arrival instructions. However, emergency medical dispatch is inherently challenged by ambiguous symptom descriptions, linguistic diversity, high cognitive load, and caller distress—factors that can compromise decision accuracy and operational efficiency [4]. Despite the implementation of standardized guide cards and dispatcher training programs, prior studies have identified limitations in triage sensitivity for time-critical conditions, such as out-of-hospital cardiac arrest, and modest predictive value for advanced life support needs [5]. Dispatchers and emergency medical services (EMS) [6] leadership increasingly view these limitations as areas where intelligent decision support could augment human judgment, particularly during complex or uncertain calls [7].

Telephone-based emergency medical dispatch is implemented somewhat differently across health systems, but shares a common core of tasks. In many North American settings, emergency medical calls are routed to public safety answering points (e.g., 9-1-1 in the United States), where trained emergency medical dispatchers use standardized protocols such as the Medical Priority Dispatch System (MPDS) to interrogate callers, assign Chief Complaints and priority codes, and provide pre-arrival instructions, while separate radio dispatchers coordinate field units [8–10]. In mainland China, callers activate EMS by dialing the dedicated “120” number, which connects to hospital-based emergency centers that both dispatch ambulances and provide medical oversight, with ambulances commonly staffed by physician–nurse–driver teams [11–13]. Thus, although staffing models and role boundaries differ—Chinese 120 systems often blur the distinction between medically trained call-takers, dispatchers, and field providers—the core interactional tasks are similar: rapid recognition and categorization of the medical problem, accurate location acquisition, and delivery of time-critical pre-arrival guidance.

The emergence of Large Language Models (LLMs) [14] has transformed the landscape of clinical natural language processing. In emergency contexts, LLMs have been used to classify call urgency [7], assist with triage decisions [15], and generate structured summaries from unstructured dispatcher-caller dialogues. Evaluation studies have shown moderate-to-strong agreement between LLM triage outputs and human paramedic decisions, suggesting early feasibility of integrating LLMs into prehospital workflows for clinical decision support [16]. Other work has demonstrated high classification accuracy of emergency versus non-emergency calls using prompt-engineered LLMs trained on call transcripts and medical scenarios [17]. More recently, multimodal applications of LLMs have emerged in the form of wearable cognitive assistants that leverage speech, vision, and biometric inputs to assist emergency responders during field operations [18].

Multi-Agent Systems (MAS) have also been explored as a complementary framework to address the coordination and operational complexity of EMS. MAS architectures enable multiple autonomous agents, representing stakeholders such as dispatch centers, ambulance teams, hospitals, or public safety systems, to collaborate in real time. These agents communicate through defined protocols to manage triage workflows, resource allocation, and route optimization, improving the efficiency and scalability of EMS systems. Earlier implementations of MAS in pre-hospital care have demonstrated benefits in task distribution and situational awareness [19, 20]. More recent work has integrated MAS with reinforcement learning to dynamically adapt responder deployments based on spatial demand patterns, enhancing response coverage and reducing travel times in densely populated urban areas [21].

The convergence of MAS and LLMs introduces an opportunity to synthesize structured coordination and natural language intelligence [22]. Similar explorations of LLMs in other high-stakes domains, such as national security applications [23], further highlight the importance of safety, accountability, and reliability in mission-critical contexts. In simulation-based EMS training, LLM-powered agents have been deployed to generate realistic, multilingual caller personas [24] and to simulate dispatcher interactions, enhancing both communication fidelity and scenario variability [25, 26]. Similarly, agent-based LLM systems have been proposed to support emergency department workflows through role-specific reasoning agents, such as triage nurses, physicians, and documentation assistants, collaboratively processing patient data in real time [27]. These architectures exemplify the potential for multi-agent LLM systems to augment human operators without replacing clinical judgment, particularly when embedded within human-in-the-loop frameworks.

Nevertheless, challenges remain in the operationalization of these technologies. LLMs are known to hallucinate factual content [28], particularly in long-context settings, and their outputs may be sensitive to prompt phrasing and domain drift. Ongoing research aims to overcome these limitations through instruction fine-tuning on curated EMS datasets and integration with retrieval-augmented generation (RAG) mechanisms that anchor model output to structured dispatch taxonomies [17, 18, 25, 27]. Similarly, MAS-based systems must ensure interoperability with existing EMS infrastructure, adherence to jurisdiction-specific triage protocols, and explainability of agent behaviors during incident review.

In this study, we extend the application of MAS and LLMs in emergency medical services by developing and evaluating a structured, LLM-powered dispatch simulation system grounded in clinically curated taxonomies and a fact commons. Our approach is distinct from prior works that also combine structured protocols with language models. EMS-BERT focuses on EMS text mining by pretraining a domain-specific transformer to extract entities and relations from static narratives, rather than generating dynamic, interactive scenarios [29]. Sim911 is closer in spirit, using an LLM caller to train human dispatchers through RAG-supported, context-controlled simulations with validation loops; however, it remains a human-in-the-loop training tool [25]. By contrast, our core novelty is a fully autonomous, multi-agent framework in which both caller and dispatcher roles are instantiated as LLM-based agents and constrained at every turn by the dispatch taxonomy and fact commons. This agent–agent paradigm can help support procedurally aligned, clinically faithful, and linguistically diverse conversations; may facilitate scaling scenario generation beyond human-centric role-play; and offers a potential basis for uses beyond training, including protocol evaluation and future decision support.

To assess system performance, we implement a hybrid evaluation strategy combining expert clinician assessments with automated metrics of linguistic quality (sentiment, readability, and politeness) and operational performance dynamics that capture how conversational efficiency, information completeness, and response pacing evolve across call phases and urgency levels. This integrated framework enables evaluation of clinical plausibility, communication fidelity, and real-time adaptive behavior under realistic dispatch workflows. The system is tested across a range of standardized emergency scenarios to evaluate realism, alignment with clinical intent, and the communicative appropriateness of AI-generated responses. Key findings include high operational quality (e.g., 94% correct external-agent contact, 97% call-back instruction, 91% advice provided), strong communication metrics (73.7% neutral sentiment, 90.4% neutral emotions, Flesch Reading Ease 80.9, and 60.0% polite with 0% impolite), and urgency-adaptive operational dynamics, with faster early information collection and shorter per-turn response times in life-critical events while achieving comparable end-of-call completeness across complaint types.

Therefore, the primary contribution of this study is threefold: (1) we construct a clinically grounded EMS taxonomy and fact commons that ground dispatcher–caller simulations; (2) we develop an LLM-based multi-agent dispatch system aligned with these structures; and (3) we design a hybrid human–algorithm evaluation framework for dispatcher conversations. Within this scope, we present the multi-agent, LLM-based system as a simulation and analysis platform for emergency medical dispatch. From structured case descriptions, the system generates end-to-end dispatcher–caller dialogues that can be examined using our taxonomy-based rubric of guidance, operational actions, and communication quality. Beyond characterizing model behavior, the platform is designed as a sandbox for stress-testing and refining call-handling protocols, and for generating structured data and design patterns that may inform future real-time decision support tools. The envisioned role of such tools is to complement and strengthen existing dispatch workflows by improving the quality and consistency of dispatcher–caller interactions, rather than to substitute for professional dispatchers.

## Methods

**Fig. 1:**
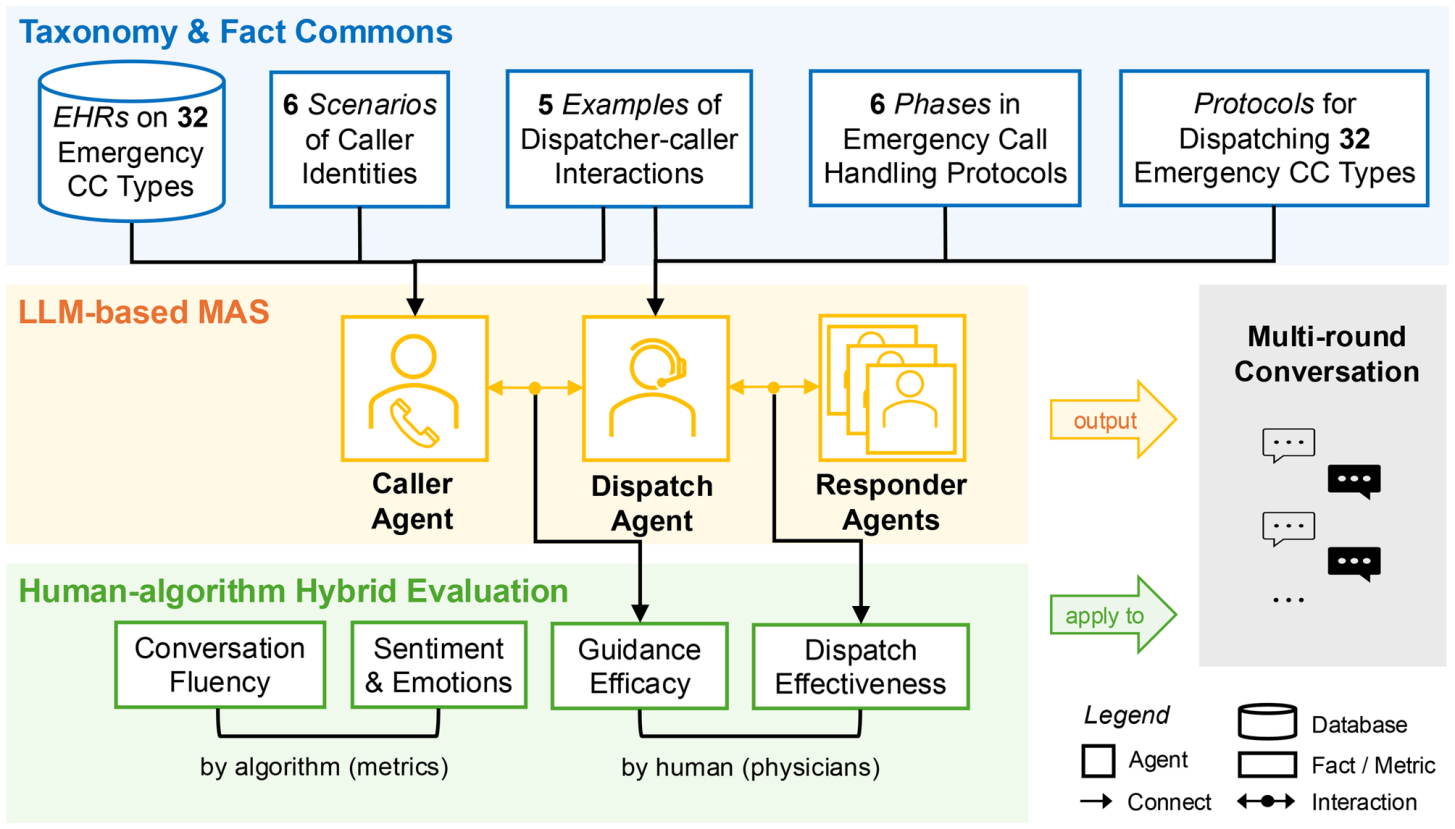
Overview of the three-phase methodology: (**a**) Taxonomy & fact commons, (**b**) LLM-based MAS, and (**c**) Human–algorithm hybrid evaluation

This study employs a systematic three-phase methodology designed to ensure clinical grounding, technical robustness, and rigorous evaluation of the proposed dispatch simulation system. As illustrated in Fig. 1, the first phase focuses on constructing a structured, standard-aligned taxonomy and fact commons that formally define 32 Chief Complaints (CCs), six caller identities, and a six-phase call-handling protocols. The second phase translates this structured knowledge into an LLM-based MAS, enabling dynamic interactions between caller, dispatcher, and responder agents. The third phase establishes a hybrid evaluation framework that combines physician adjudication of guidance efficacy and dispatch effectiveness with automated assessments of communication quality (conversation fluency, sentiment/emotions, readability, and politeness) and transcript-derived operational performance dynamics capturing information-collection efficiency, completeness, and simulated pacing across call phases and complaint urgency. Together, these phases provide an integrated pathway for developing and validating AI agents in emergency medical services.

### Data

We utilized 50 validated, de-identified patient records derived from the MIMIC-III database [30], spanning diverse demographics (age 19–100; 52% male) and emergency admission types. For this proof-of-concept simulation, we did not use the full MIMIC-III cohort; instead, we manually selected a small set of adult emergency admissions with sufficient structured information to create case vignettes covering our 32 Chief Complaint categories.

Each case was further enriched via a Neo4j knowledge graph to integrate complex medical histories and admission contexts. Clinical presentations are organized by an EMDPRS-based emergency taxonomy comprising 32 CCs, grouped into Individual Complaints, Traumatic Incidents, and Life-Critical Events. To simulate realistic interactions, we predefined six caller identity types (Patient, Bystander, Family/Associate, Multiple Callers, Involved Party, Limited-Proficiency Caller) and—for this dataset—assigned exactly two identities to each patient case to generate dialogues; with *N* cases this yields 2*N* call scenarios, so with fifty cases we obtain one hundred synthetic dispatcher–caller dialogues. Agent communication patterns and conversational tone were calibrated using five de-identified, training 911 call exemplars from dispatcher educational materials. These examples were used only to inform taxonomy refinement and prompt design and are not themselves part of the analyzed dataset. No operational 9–1-1/120 logs, identifiable call recordings, or real call transcripts were accessed; all evaluated dialogues in this study are fully synthetic and generated from the curated scenarios and fact commons. A detailed summary of these data components is provided in Table 1.

**Table 1:** Data statistics

Data Component	Metric	Value / Distribution
**Patient Records**	Sample Size	50 unique patients
	Age Range	19 - 100 years
	Gender Distribution	52% Male (*n* = 26), 48% Female (*n* = 24)
**Emergency Scenarios**	Individual Chief Complaint	27 cases
	Traumatic Incident Type	15 cases
	Time/Life-Critical Event	8 cases
**Conversational Diversity**	Caller Identity Types	6 distinct types
	Caller Identities per Patient	2 - 6 types
	Real-World Call Transcripts	5 full transcripts

To operationalize limited proficiency within the confines of English-only simulation, we additionally conducted a controlled comparison between fluent and limited-English-proficiency caller constraints (40 calls per condition) using the same scenarios and evaluation rubric (Supplementary Methods S7).

### A taxonomy and fact commons for emergency medicine

We developed a clinically grounded taxonomy and fact commons to systematically organize the diverse scenarios encountered in emergency medical dispatch. This structured knowledge base underpinned our simulation system by ensuring that language model agents operated within clinically grounded and procedurally accurate representations of emergency calls. To construct the taxonomy, we identified 32 distinct Chief Complaints (CCs) derived from the Emergency Medical Dispatch: National Standard Curriculum published by the National Highway Traffic Safety Administration (NHTSA) [31, 32] and the Health Resources and Services Administration (HRSA), with additional guidance from the Maternal and Child Health Bureau. These CCs spanned a wide range of emergency types, including medical, traumatic, environmental, and obstetric events, and were selected for their representativeness and relevance to dispatcher workflows [33].

For each CC, we curated structured information to support both simulation and interpretation. The detailed field schema, curation principles, and full categorization of this taxonomy are available in Supplementary Method S1. This included the clinical background and typical etiologies, the most frequently reported symptoms and situational patterns, standard pre-arrival instructions, special considerations for pediatric and maternal cases, and integration of relevant local and national dispatch protocols (Supplementary Table S1). Together, these elements ensure clinical fidelity and provide a structured fact commons that enables agents to respond in ways consistent with best practices in emergency medicine.

To simulate realistic emergency interactions, we further designed six prototypical caller identities reflecting the diversity of real-life dispatch scenarios [34]:

1. **Patient**: the individual, who may be acutely symptomatic and have limited capacity to communicate;
2. **Bystander**: a third-party bystander, present but unfamiliar with the patient’s background;
3. **Family/Associate**: a family member or close associate, often introducing emotional urgency;
4. **Multiple Callers**: multiple concurrent callers, potentially providing conflicting information;
5. **Involved Party**: an individual possibly responsible for the emergency; and
6. **Limited-Proficiency Caller**: a caller with constrained functional communication, including reduced fluency, limited vocabulary, or difficulty understanding complex questions.

These identities were grounded in clinical narratives drawn from the MIMIC-III electronic health record dataset [35], enabling the system to reflect realistic expressions of symptoms, caller behaviors, and information quality.

In parallel, we encoded the standard progression of an emergency call using a six-phase protocol widely adopted in dispatcher training and operations [33]. While this structure is formalized in the U.S. Emergency Medical Dispatch National Standard Curriculum, the underlying steps are broadly consistent with Chinese 120 practice, where dispatch guidance similarly emphasizes confirming the caller’s location and contact details, eliciting the Chief Complaint and key symptoms, prioritizing urgency, and providing pre-arrival instructions before handoff to the ambulance team:

1. **Initial Intake**: where call handlers collect essential information such as location, patient identity, and nature of the emergency;
2. **Scene Condition Assessment**: eliciting contextual details to evaluate environmental safety and condition severity;
3. **Dispatch**: initiating appropriate response units based on collected information;
4. **Provision of Real-Time Updates**: as new details emerge;
5. **Delivery of Pre-arrival Instructions**: such as cardiopulmonary resuscitation (CPR) and bleeding control to stabilize the patient prior to responder arrival; and
6. **Call Closure**: formally concluding the interaction once professional responders assume care.

### An LLM-based agentic EMS dispatch system

**Fig. 2:**
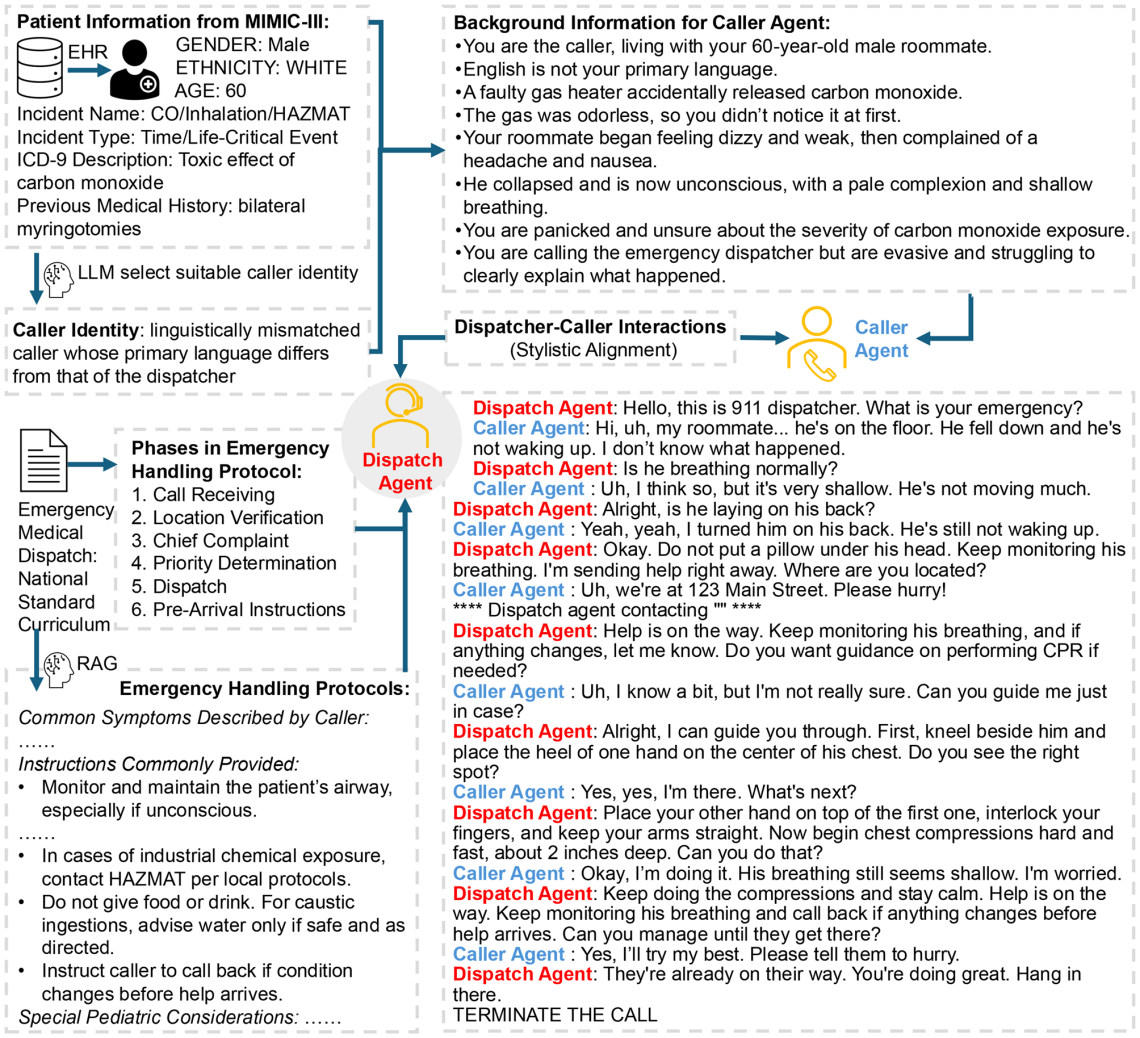
Schematic overview of the LLM-based multi-agent emergency medical dispatch (EMD) simulation system. Patient information derived from MIMIC-III electronic health records (EHR) seeds realistic caller backgrounds and identities. Knowledge from the Emergency Medical Dispatch National Standard Curriculum—including Chief Complaint taxonomy, handling protocols, and call-handling phases—grounds both caller and dispatcher agents. The pipeline illustrates caller scenario generation and identity selection, dispatcher–caller interactions with turn-level chief-complaint classification and protocol retrieval, optional escalation to auxiliary agents, and delivery of pre-arrival instructions, as exemplified by the representative call transcript

Building on the structured taxonomy and fact commons described in Sect. 2.2, our second major contribution is the development of a multi-agent emergency medical dispatch (EMD) simulation system powered by LLMs. As illustrated in Fig. 2, the system integrates structured medical knowledge, realistic caller identities, and standardized dispatch protocols to simulate end-to-end emergency call scenarios. Two core agents, a caller agent and a dispatcher agent, interact dynamically within the AutoGen framework [36] to reproduce the real-world 911 calls. Responder agents are invoked by the dispatcher agent only when needed—for example, when an ambulance is dispatched or a handoff to the receiving team is required—to simulate downstream clinical actions and complete the end-to-end workflow. The specific component versions, including the core LLMs and software frameworks, are documented in Supplementary Table S2 to ensure reproducibility.

In brief, each simulated call unfolds as a turn-by-turn interaction between the dispatcher and caller agents. At every dispatcher turn, the system (i) updates a running summary of the call, (ii) classifies the Chief Complaint and priority using the EMS taxonomy, and (iii) retrieves only incident-specific rules and pre-arrival instructions from the shared clinical knowledge base before generating a single focused question or instruction in plain language. The caller agent then responds using only information available in the scenario description and prior turns, following constraints that enforce a layperson communication style and prevent access to hidden “god’s-eye” knowledge. If the available information is insufficient to determine a specific protocol branch, the dispatcher agent uses a predefined “Lack of Information” fallback rather than guessing.

To reduce unsafe or unrealistic behavior, we implement several hard-coded safeguards. Dispatcher outputs are restricted to one action per turn, must follow safety-first ordering (scene safety and life threats before secondary details), and are explicitly prohibited from offering medical diagnoses or off-protocol treatment advice. All calls must end with a standardized safety-net closure (e.g., advising the caller to recontact EMS if the situation changes). Scenario-level exclusion rules further prevent implausible or unsafe setups (such as unconscious callers or self-incriminating perpetrators). Full architectural details, prompt templates, and safety rules are provided in Supplementary Methods S2–S5 and Supplementary Tables S3–S6.

A well-known limitation of LLMs is their tendency to generate plausible but inaccurate or fabricated responses, commonly referred to as hallucinations [37]. To mitigate this risk, both agents are grounded in the fact commons introduced in Sect. 2.2. By constraining the generative process with structured, clinically relevant knowledge at each conversational turn, the system minimizes misinformation and enhances clinical realism.

The caller agent is designed to simulate diverse and naturalistic emergency calls. For each case, we generate a narrative background scenario rather than directly exposing structured patient data. Using profiles derived from MIMIC-III, the LLM selects the most contextually appropriate caller identity from six predefined roles—patient, bystander, family member or associate, multiple callers, potentially responsible individual, or linguistically mismatched caller—while excluding logically inconsistent options (e.g., an unconscious patient acting as the caller). The generated scenario includes contextual details such as caller–patient relationship, setting (e.g., home or public space), time of day, and observable symptoms (see Supplementary Table S7 for sampling variables). This approach achieves two objectives: it ensures realistic alignment of caller identity with clinical context and avoids overly precise or implausibly clinical descriptions that would not occur in real conversations. The construction workflow, sampling variables, and critical safety-first consistency rules governing the Caller Agent generation are detailed in Supplementary Method S2. To further approximate authentic call dynamics, the caller agent utterances are calibrated with linguistic samples from five real 911 transcripts, allowing the system to reproduce the ambiguity, urgency, and emotional tone typical of emergency calls.

The dispatcher agent is initialized with standardized Emergency Call Handling Protocols and the same authentic call transcripts used to calibrate the caller agent. At each conversational turn, a lightweight classifier reviews the dialogue history and assigns one of the 32 CCs categories defined in the taxonomy, or a fallback category named ‘Lack of Information’ if insufficient evidence is available. Once a CC is determined, we employ a RAG mechanism. This process dynamically retrieves relevant procedural and clinical instructions specific to the identified CC from our knowledge base and integrates them into the dispatcher agent’s prompt. This ensures structured questioning, appropriate red-flag coverage, and accurate pre-arrival guidance. All agents were instantiated using the GPT-4o model accessed via API. We selected GPT-4o as a representative frontier LLM based on three practical criteria: strong published performance on complex language tasks, native support for tool/function calling required by our multi-agent design, and a cost profile compatible with running 100+ end-to-end simulations. The system’s turn-level RAG loop and the full prompt library, including global hard constraints designed to ensure safe and coherent interactions, are described in Supplementary Method S3 and Supplementary Method S4. The full prompts governing the dispatcher agent, caller agent, CC classifier, and RAG injection template are provided in Supplementary Tables S3–S6.

In addition, the system supports multi-agent escalation and external tool integration. Each CC protocol is annotated with potential auxiliary resources, such as the EMDPRS or external agencies (e.g., police, fire services). Upon CC confirmation, the dispatcher can invoke these resources through function calls. In this study, responses from auxiliary agents were mocked by LLMs to maintain dialogue flow, but the modular design allows plug-and-play integration with real APIs or agent logic in future deployments.

Taken together, the LLM-based MAS operationalizes the end-to-end workflow depicted in Fig. 2: structured case metadata and EHR-derived narratives seed realistic caller scenarios; the dispatcher iteratively classifies CCs and retrieves protocol-bound decision steps; pre-arrival instructions are delivered when criteria are met; and, when applicable, function calls trigger auxiliary agents (e.g., EMDPRS, police, fire). By unifying grounded medical knowledge, narrative caller modeling, dynamic CC-conditioned prompting, and modular escalation, the framework yields high-fidelity simulations that mirror real 9–1-1 interactions while maintaining clinical safety. This platform is therefore best viewed as a controlled simulation and analysis environment, providing a plug-and-play pathway for future work on protocol refinement, decision-support interface design, and, where appropriate, integration with live EMS systems.

### Human–algorithm hybrid evaluation framework

We evaluated the AI-enhanced dispatch system using a hybrid framework that integrates structured human expert assessment with automated transcript-based metrics. This design targets three complementary goals: *clinical validity*, assessing whether the system adheres to emergency medical dispatch protocols and provides safe, appropriate guidance; *communicative robustness*, assessing whether the system maintains clarity, calmness, and professionalism under stressful conditions; and *operational performance dynamics*, assessing how efficiently and adaptively the system collects critical information and progresses through call phases under simulated time pressure.

#### Integrated assessment

Human and automated evaluations provide complementary and non-redundant perspectives. Human ratings capture clinical appropriateness, protocol adherence, and practical utility from an expert standpoint. Automated communication metrics quantify tone and accessibility at scale (e.g., sentiment/emotions, readability, and politeness), providing a systematic check on interactional quality. Operational performance dynamics further quantify process-level behavior—such as information-collection efficiency, completeness, and simulated pacing across call phases and complaint urgency—linking what the system says to how the call unfolds. Together, these components form an integrated framework for validating clinical, communicative, and operational performance to support iterative refinement and inform future deployment studies.

#### Human evaluation

Human evaluation was organized into three domains: **Guidance Efficacy**, **Dispatch Effectiveness**, and **Interactional Alignment**. Guidance Efficacy captured physicians’ judgments of the clarity and appropriateness of pre-arrival advice, defined as non-diagnostic instructions given to callers (e.g., positioning, monitoring, immediate safety actions, and when to recontact EMS or seek urgent care). Dispatch Effectiveness focuses on the operational quality of the call: whether questioning is proportionate and appropriately targeted to identify the main problem and red-flag features, consistent with work on telephone triage accuracy and urgency allocation [38, 39]; whether the caller’s location is clearly elicited and confirmed, given that difficulties determining location are a major contributor to delayed EMS response and that mobile- and handset-based location systems significantly shorten response times [40–42]; whether the right additional services (such as police, fire, or poison control) are contacted when indicated, in line with guidance that positions poison control and other agencies as integral components of the emergency-response chain [43, 44]; and whether explicit safety reminders are given (for example, to call back if the situation changes), reflecting the “safety netting” literature in primary and remote care, which emphasizes advising patients about red-flag symptoms, when to seek further help, and how to re-access services as a core strategy to mitigate diagnostic and triage risk [45, 46]. These aspects align with established concerns in telephone triage and dispatch about balancing thorough information gathering, accurate localization and categorization, and timely resource mobilization.

The Interactional Alignment domain is informed by conversation-analytic work on emergency and helpline calls, which treats call handling as an interactional accomplishment rather than a simple information transfer. Drawing on Whalen and Zimmerman’s analyses of “practical epistemology” in citizen calls to the police [47], Tracy’s work on frame clashes [48] and resistance to standardized questioning, and Shaw and colleagues’ studies [49] of managing distress and stance in telephone support, we evaluate (i) how well the dispatcher aligns frames and explains protocol-driven questioning, (ii) how appropriately they establish and use the caller’s epistemic access to the event, and (iii) how effectively they manage interactional trouble such as resistance, misunderstanding, or misalignment of roles. To explicitly probe misalignment and repair within the same interactional framework, we conducted a controlled trigger perturbation study in which the caller agent was instructed to introduce specific trouble sources during the call (e.g., resistance to questioning/instructions, role mismatch as a bystander with limited knowledge, or initially vague everyday descriptions requiring negotiated shared framing), compared against a no-trigger baseline (Supplementary Methods S8). All other elements—including clinical scenarios, dispatcher policy, and evaluation rubric—were held constant. Together, these three domains allow us to assess both the substantive outcomes of the call (guidance and actions) and the interactional work through which those outcomes are achieved. The structured questionnaire is shown in Table 2.

**Table 2:** Overview of evaluation questionnaire

Category	Sub-Category	Question	Answer Type
Guidance Efficacy	Advice given	Did the Dispatcher provide advice to the Caller?	Binary (Yes/No)
Guidance Efficacy	Satisfaction with amount of advice	Was the amount of advice provided by the Dispatcher adequate?	Ordinal (1–5)^1^
Guidance Efficacy	Helpfulness of advice, if given	Was the advice given by the Dispatcher helpful in assisting the Caller during the emergency?	Ordinal (1–5)^1^
Dispatch Effectiveness	Number of questions asked and answered	Was the number of questions asked and answered between the Dispatcher and Caller reasonable?	Ordinal (1–5)^1^
Dispatch Effectiveness	Relevance of questions asked and answered	Did the Dispatcher ask relevant questions to identify the medical issue?	Ordinal (1–5)^1^
Dispatch Effectiveness	Contact the correct potential other agents	Did the Dispatcher successfully contact the correct potential other agents?	Binary (Yes/No)
Dispatch Effectiveness	Told to call back if necessary	Did the Dispatcher advise the Caller to call back if necessary?	Binary (Yes/No)
Dispatch Effectiveness	Location obtained	Did the Dispatcher clearly elicit and confirm the Caller’s location (including necessary details to dispatch help)?	Binary (Yes/No)
Interactional Alignment	Frame Alignment & Information Management	Did the Dispatcher clearly explain why they needed to ask multiple questions / follow a protocol before sending help?	Ordinal (1–5)^1^
Interactional Alignment	Epistemic Appropriateness: Establishing caller’s knowledge basis	Did the Dispatcher explicitly establish how the Caller knows about the problem (e.g., direct witness, hears screams, told by others)?	Binary (Yes/No)
Interactional Alignment	Epistemic Appropriateness: Shared medical problem framing	By the end of the call, was there a clear, medically meaningful shared understanding of the main problem (e.g., “unconscious adult,” “possible stroke,” “severe breathing difficulty”)?	Ordinal (1–5)^1^
Interactional Alignment	Interactional Trouble Management: Handling resistance to questions / instructions	When the Caller resisted questions or instructions, did the Dispatcher respond in a way that de-escalated the interaction (e.g., calm repetition, brief justification) rather than escalating conflict?	Ordinal (1–5)^1^
Interactional Alignment	Interactional Trouble Management: Alignment with caller’s stance and role	Did the Dispatcher appropriately acknowledge the Caller’s stance and role (e.g., direct victim, bystander, third-party reporter), in a way that supports collaboration?	Ordinal (1–5)^1^

*Note.* ^1^ Anchors for all ordinal items: 1 = strongly dissatisfied, 2 = dissatisfied, 3 = acceptable, 4 = satisfied, 5 = very satisfied

**Fig. 3:**
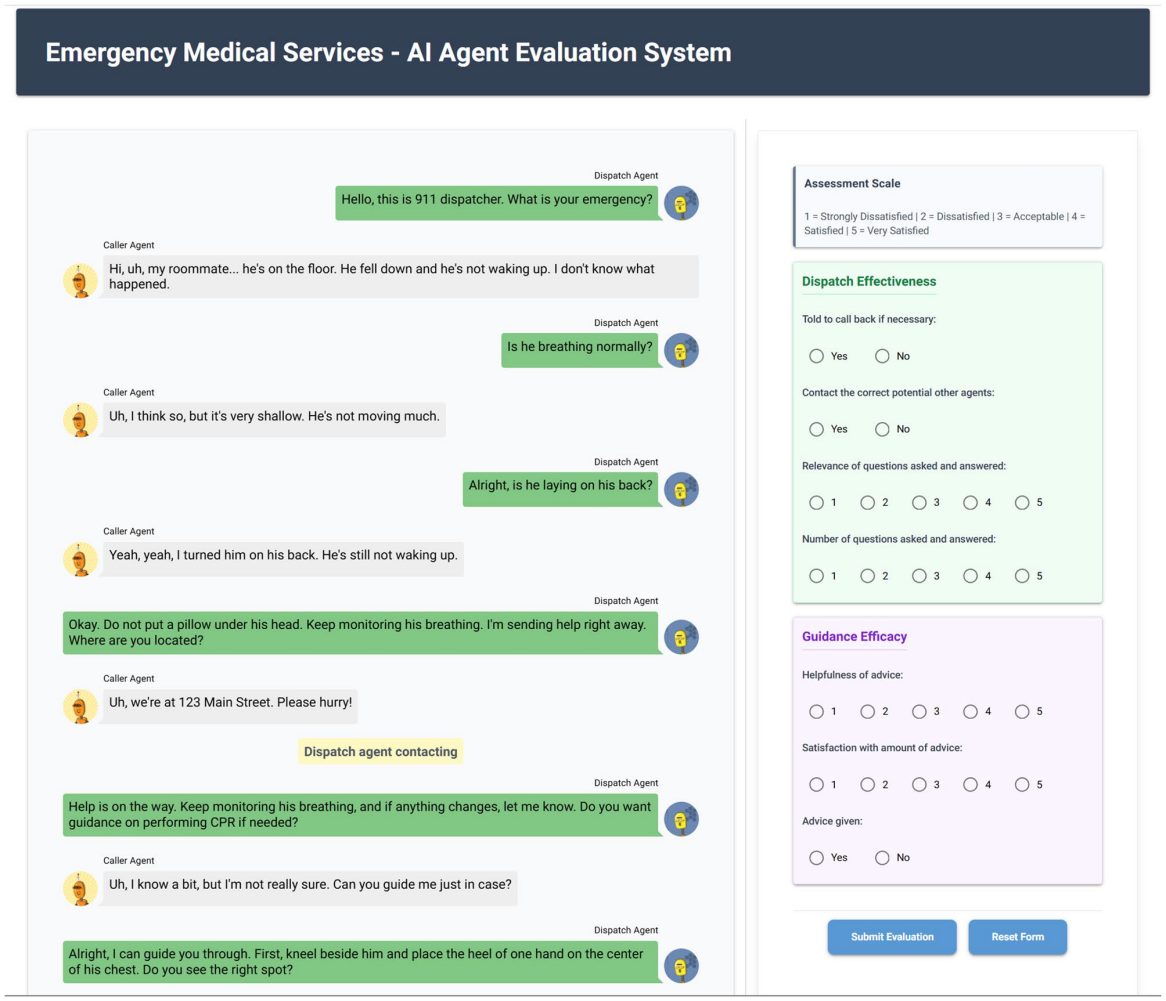
Evaluation interface of the AI agent system

We generated 100 simulated emergency call scenarios covering diverse CCs and caller profiles. Four licensed physicians who completed emergency medicine training/rotations and have experience with acute triage served as expert raters. To reduce single-system bias, we recruited emergency physicians with prehospital experience in both United States 9–1-1 and Chinese 120 systems to rate a sample of dialogues using the same rubric, so that our human evaluation reflects perspectives from two distinct EMS traditions. Each rater independently reviewed an assigned subset of cases using the questionnaire, while 20 cases were annotated by all four physicians to assess inter-rater reliability. Raters were blinded to model version and trained on a calibration set to standardize interpretation. The evaluation interface, as shown in Fig. 3, consist of two panels: the left panel displays the dialogue between the caller agent and the dispatch agent, while the right panel contains the standardized evaluation options. Physicians submit their assessments through this interface.

#### Controlled communication ablations

To examine how specific communication strategies influence information elicitation and interactional outcomes within a fixed clinical scenario, we conducted a controlled ablation in which only the dispatcher’s question-framing policy was varied while keeping the underlying case vignettes, protocols, and evaluation rubric unchanged (Supplementary Methods S6). We compared multiple framing styles that differ in cognitive load and epistemic steering (e.g., single-focus sequential questioning versus bundled/overloaded questioning, as well as jargonized and medicalized/leading formulations). Each style was evaluated on the same rubric-based outcomes and operational markers reported in the main study; full experimental setup and results are provided in Supplementary Methods S6.

#### Algorithmic evaluation

To complement expert judgment, we assessed four communication-related dimensions: **Sentiment Analysis** (overall evaluative tone), **Emotion Classification** (dominant affective state), **Readability Assessment** (linguistic simplicity under stress), and **Politeness Evaluation** (respectful, professional phrasing). These choices are motivated by evidence that the manner of communication can strongly influence cooperation, comprehension, and trust in emergency and clinical encounters, above and beyond the factual content. Studies of telephone calls to emergency medical services show that callers’ emotional states and the way call-takers manage affect are associated with out-of-hospital cardiac arrest recognition and the successful delivery of dispatcher-assisted CPR; calmer, more engaged communication is linked to better caller cooperation and adherence to instructions [50, 51]. Quantifying sentiment and dominant emotion for dispatcher turns therefore provides a scalable proxy for whether responses remain affectively balanced—neither unduly cold nor escalating panic—and appropriately calibrated to the crisis context.

Readability is a longstanding concern in health communication: emergency department discharge instructions and written patient materials are frequently written above recommended reading levels, and lower readability is associated with poorer comprehension and weaker adherence to instructions [52–54]. In an emergency call, where callers may be distressed, distracted, or have limited health literacy, ensuring that spoken instructions are as simple and easy to parse as possible is critical. Finally, perceived respect and dignity, as well as patient-centred communication, have been linked to higher trust and better patient experience in clinical settings [55, 56]. An explicit politeness score allows us to detect and avoid responses that are unnecessarily brusque or face-threatening, while still permitting firm, time-critical directives when needed. Together, these four automated metrics provide complementary, theory-grounded indicators of communication quality that augment expert ratings of clinical and operational performance. Categories, taxonomies, and models are summarized in Table 3, and specific model checkpoints and specifications are detailed in Supplementary Table S8.

**Table 3:** Algorithmic evaluation metrics and models

Category	Typology	Taxonomy	Model / Calculation
Sentiment Analysis	Multi-Class	Positive, negative, and neutral	RoBERTa-base
Emotion Classification	Multi-Class	Disgust, joy, sadness, anger, fear, surprise, and neutral	E-DistilRoBERTa
Readability Assessment	Continuous	0–100	Flesch Reading Ease
Politeness Evaluation	Multi-Class	Polite, somewhat polite, neutral, impolite	Bert-base

Sentiment was classified using RoBERTa-base fine-tuned on TweetEval (negative / neutral / positive) [57], and emotion classification followed Ekman’s taxonomy via E-DistilRoBERTa trained on the EmoEvent corpus [58] and supplementary datasets (seven categories: disgust, joy, sadness, anger, fear, surprise, neutral) [59]. Politeness was evaluated with a BERT-base classifier trained on synthetic customer-service interactions (polite / somewhat polite / neutral / impolite), and readability was measured using the Flesch Reading Ease score (0–100; higher values indicate greater accessibility). These pretrained models were selected because they have been widely validated on large-scale conversational text (e.g., social media and customer-service dialogues) and provide robust, off-the-shelf tools for coarse-grained profiling of sentiment, emotion, and politeness. Our goal was not to make case-level clinical inferences from these labels, but to characterise broad differences in language use between dispatcher and caller across many simulated calls. We therefore treat these outputs as approximate indicators of affective tone and communication style in English, acknowledging that the models are not specifically trained on emergency call transcripts; developing EMS-specific, fine-tuned classifiers is an important direction for future work.

Because callers in emergency contexts are often highly distressed, maintaining clarity and emotional appropriateness is critical. At the same time, conversation-analytic studies emphasize that not all callers display overt emotion; an apparently calm or affectively flat delivery can itself become a source of communicative misalignment, requiring additional interactional work to establish urgency and shared framing [60, 61]. Overly emotional responses may escalate anxiety, whereas unclear or overly technical instructions risk compromising patient safety. Algorithmic evaluation thus complements expert review by providing a scalable check on communication quality alongside clinical safety.

#### Operational performance dynamics evaluation

To assess the system’s efficiency and responsiveness under simulated time pressure, we conducted a post-hoc analysis based on the dialogue transcripts. A quantitative assessment of operational performance across 100 cases, including distributions for information completeness, response timeliness, and guidance accuracy, is presented in Supplementary Fig. 2. The detailed scoring methodology for these metrics is defined in Supplementary Table S10. It is important to note that the timeline used for this analysis is a simulation calculated from the number of utterances, not a measure of real-world computational latency. The core metric, the “Information Collection Efficiency Score,” is calculated based on the proportion of predefined critical entities (e.g., location, consciousness) successfully elicited by the agent for a given scenario.

#### Statistical analysis

Given skewed category prevalences and multiple raters, we used Gwet’s AC1—less sensitive to prevalence/marginal imbalance than *κ*—to estimate inter-rater reliability (95% CI). We summarized study outcomes with descriptive statistics (frequencies and percentages) for both binary and ordinal ratings. To examine between-rater differences, we used one-way Analysis of Variance (ANOVA) for ordinal ratings, treating them as approximately continuous and checking normality and homoscedasticity; and Pearson’s Chi-squared tests for binary outcomes (Fisher’s exact test when expected counts were < 5). Two-tailed tests were used with *α* = 0.05; *p*-values are nominal with no adjustment for multiple comparisons. The same inferential procedures were applied to the supplementary ablations (Supplementary Method S7–S8), with proportions compared by Fisher’s exact/chi-squared tests and rating-scale outcomes compared using one-way ANOVA or two-sample tests as appropriate (Supplementary Method S7–S8).

## Results

This section presents findings from our hybrid evaluation framework. We first report the human evaluation results, including inter-rater reliability and the system’s performance on Guidance Efficacy and Dispatch Effectiveness; we then present algorithmic assessments of the agent-generated dialogue (sentiment/emotions, readability, and politeness) alongside transcript-derived Operational Performance Dynamics that quantify information-collection efficiency, completeness, and simulated pacing across call phases and complaint urgency.

### Human evaluation

#### Inter-rater reliability

To validate the consistency of our human evaluation, we first assessed inter-rater reliability on the 20 cases annotated by all four physicians. The analysis yielded a Gwet’s AC1 score greater than 0.70 across all evaluation metrics, indicating substantial agreement among the physicians and supporting the robustness of our assessment framework.

#### System performance on dispatch, guidance Efficacy, and interactional Alignment

**Fig. 4:**
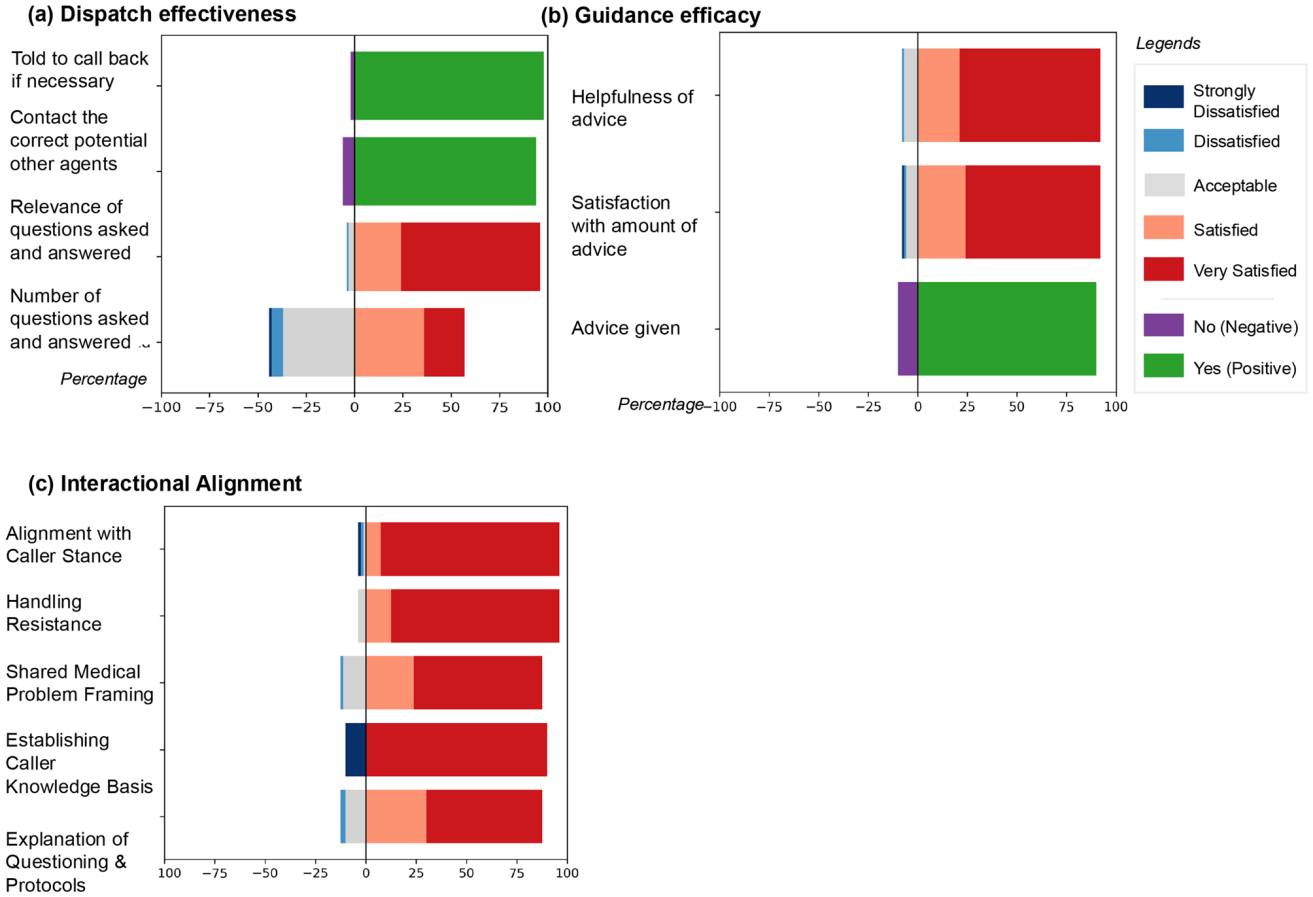
Evaluation results in (**a**) dispatch effectiveness and (**b**) guidance efficacy. Bars represent the percentage distribution of binary (yes/No) and ordinal (1–5) responses

The system demonstrated strong performance across Dispatch Effectiveness and Guidance Efficacy as rated by the physicians. As shown in Fig. 4(a), the system achieved high Dispatch Effectiveness: in binary assessments, it successfully identified and contacted the correct potential other agents in 94% of cases and provided advice to call back if necessary in 97% of cases. For ordinal metrics, the relevance of questions asked was predominantly rated highly (scores of 4 or 5). Notably, the number of questions asked received a neutral rating (score of 3) in 37% of cases, suggesting an area for further calibration. Guidance Efficacy was rated exceptionally high across all metrics (Fig. 4(b)). The dispatcher agent provided advice in 91% of scenarios where it was deemed necessary, and both the satisfaction with the amount of advice and the helpfulness of the advice received were overwhelmingly positive, with the majority of scores being 4 or 5. In addition, the conversation-analytic informed Interactional Alignment domain indicated consistently strong interaction management (across rated cases, *n* = 80). The dispatcher was rated highly for aligning with the caller’s stance and role (scores 4–5 in 77/80, 96.3%), handling resistance without escalation (4–5 in 77/80, 96.3%), and achieving a clear shared medical problem framing by call end (4–5 in 70/80, 87.5%). Raters also frequently endorsed the dispatcher’s explanation of protocol-driven questioning (4–5 in 70/80, 87.5%). Finally, the dispatcher explicitly established the caller’s knowledge basis in 72/80 cases (90.0%), supporting effective epistemic grounding of the interaction. In additional framing ablations, single-focus sequential questioning produced the most consistently favorable guidance, completeness, and alignment ratings, whereas bundled/overloaded questioning degraded interactional alignment and perceived efficiency (Supplementary Methods S6). In trigger perturbations designed to induce interactional trouble, we observed statistically significant between-condition differences in trouble management and response timeliness, with the resistance condition producing the largest alignment and pacing penalties (Supplementary Methods S6).

#### Statistical analysis of rater disagreement

To investigate potential variability among raters, we conducted statistical analyses to compare the physicians’ annotations. As summarized in Supplementary Table S9, we found statistically significant differences among raters on two metrics: ‘Relevance of Questions Asked and Answered’ (ANOVA, F = 4.301, *p* = 0.007) and ‘Contact the correct potential other agents’ (Chi-squared test, *χ*^2^ = 16.0, *p* = 0.001). For all other metrics, no statistically significant differences were observed, indicating a high degree of consensus. A post-hoc review of individual rater scores revealed that the difference in ‘Relevance of Questions’ was primarily driven by one rater (Physician #4) providing consistently lower ratings compared to another (Physician #1).

### Algorithmic evaluation

**Fig. 5:**
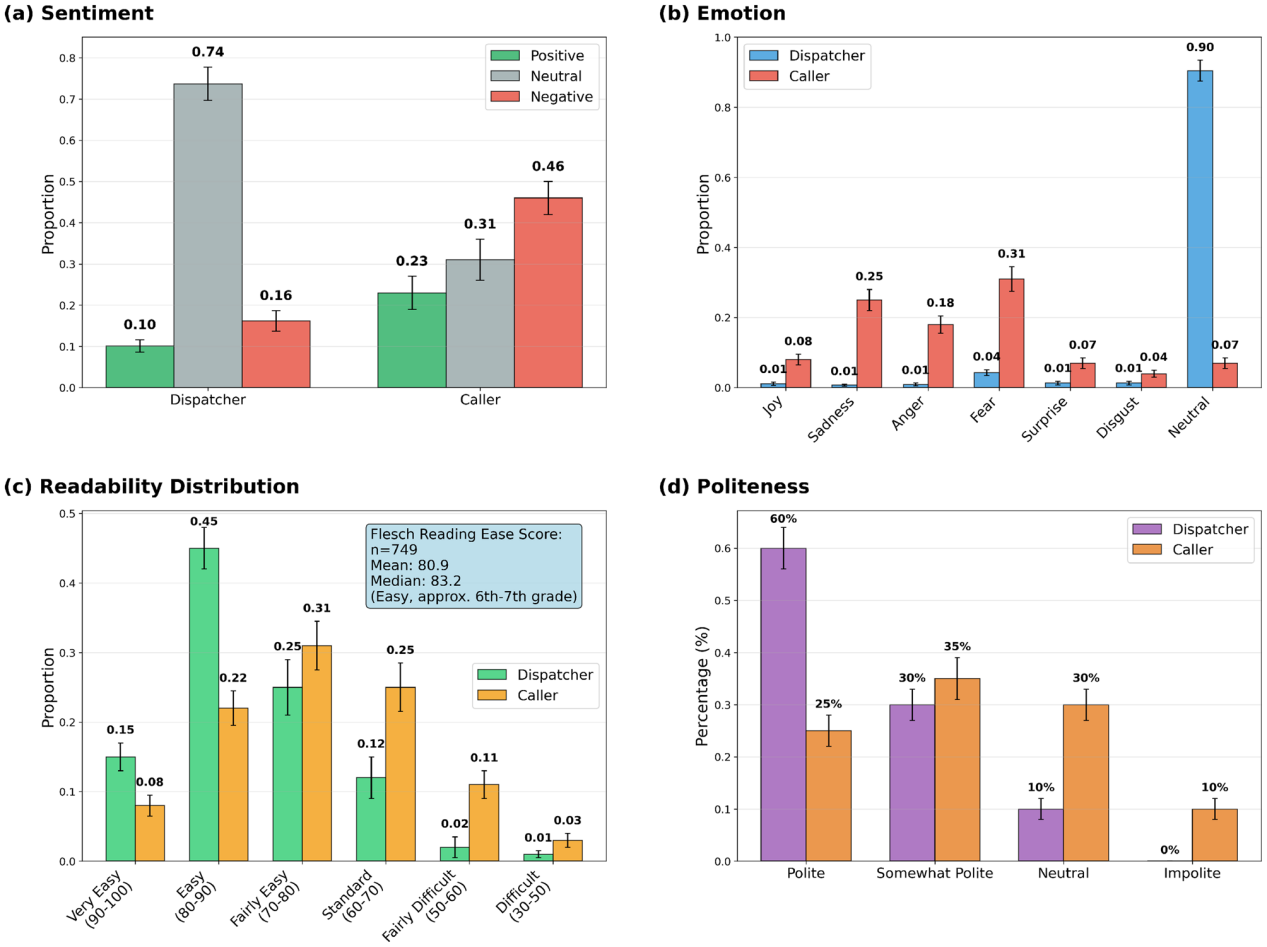
Algorithmic evaluation comparing dispatcher and caller dialogue profiles across four key metrics: (**a**) sentiment distribution, (**b**) emotion classification, (**c**) readability based on Flesch Reading Ease scores, and (**d**) politeness levels

The algorithmic evaluation provided quantitative insights into the communication quality and affective dynamics of the dispatcher-caller dialogues. The analysis revealed a clear and appropriate distinction between the dispatcher’s professional, calm profile and the caller’s simulated distress, as visualized in Fig. 5.

#### Sentiment and emotion analysis

Analysis of the agent responses revealed a stark contrast in their affective profiles. As shown in Fig. 5a, the dispatcher agent maintained a predominantly neutral sentiment (74%), appropriate for professional emergency communication. In contrast, the caller agent’s dialogue was primarily negative (46%) and less neutral (31%), effectively simulating the emotional state of a person in an emergency. This emotional gap was further confirmed by emotion classification (Fig. 5b), where the dispatcher’s output was overwhelmingly neutral (90.4%). Conversely, the caller exhibited significant levels of fear (31%) and sadness (25%), emotions consistent with the urgency and seriousness of the scenarios. We note that real EMS calls include a wider range of affective displays, and a perceived absence of emotion can also generate interactional trouble; our current caller agent was primarily tuned to represent distressed presentations [60, 61].

#### Readability and politeness

The readability of the dispatcher’s messages was high, suggesting instructions were clear and accessible to a distressed caller. The messages achieved a mean Flesch Reading Ease score of 80.9, equivalent to a 6th-7th grade reading level. As shown in Fig. 5c, the distribution of the dispatcher’s language peaks in the “Easy” category (43%), while the caller’s language was naturally more varied and complex. Politeness evaluation confirmed a high standard of professional communication from the dispatcher (Fig. 5d). A majority of its responses were classified as “polite” (60%), with an additional 30% as “somewhat polite,” and importantly, no responses were deemed “impolite”. This demonstrates a consistent adherence to respectful and supportive communication norms, which is critical in high-stakes settings.

### Operational performance dynamics

**Fig. 6:**
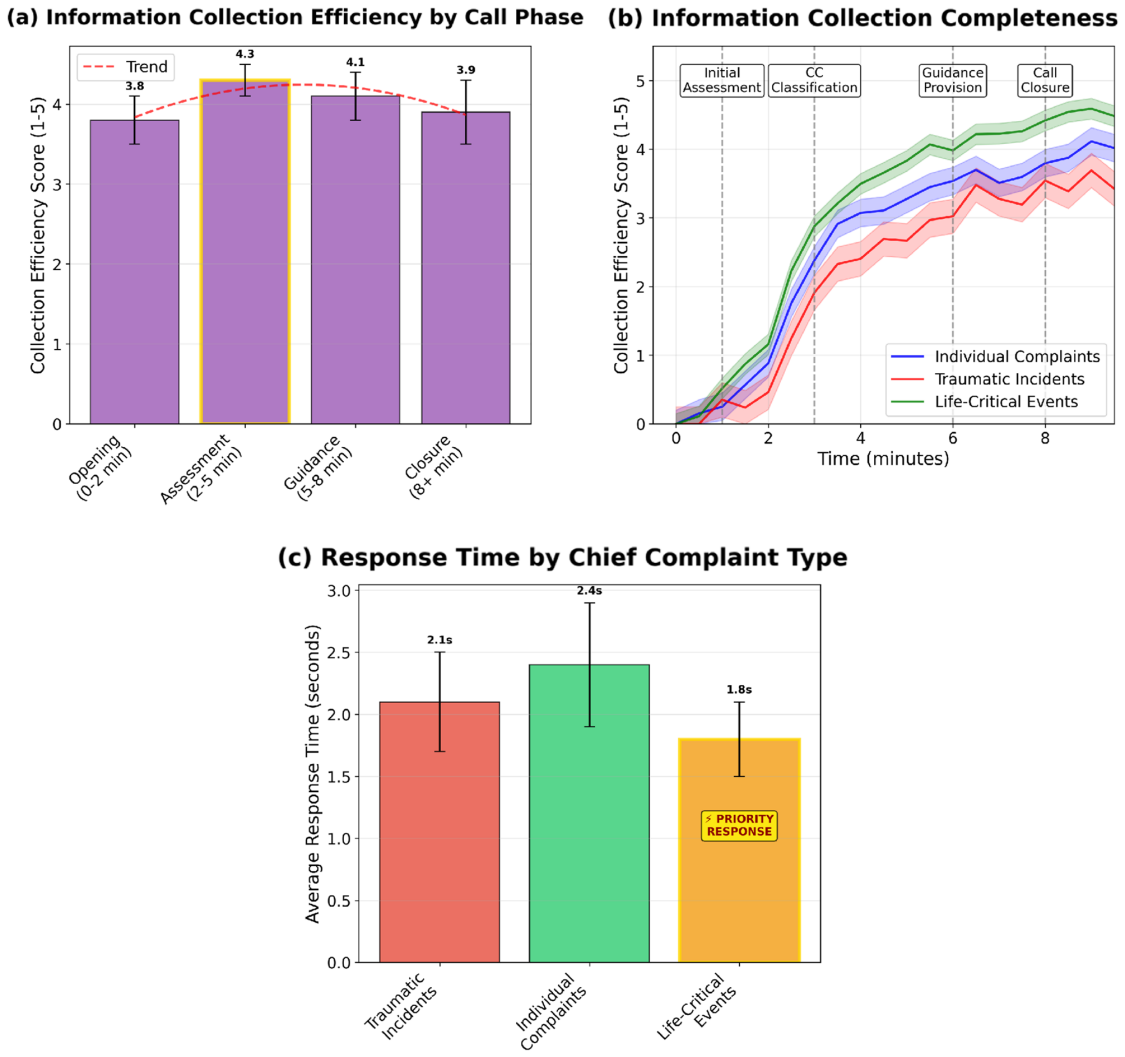
Post-hoc analysis of information collection and response dynamics. (**a**) The information collection efficiency score (1–5) is highest during the “assessment” phase of the call, aligning with the core task of that period. (**b**) The trace of collection completeness over simulated time shows that the system gathers information most rapidly for “life-critical events” (green line), demonstrating effective prioritization. (**c**) The average simulated response time is fastest for “life-critical events” (1.8s), confirming the system’s ability to adapt its conversational strategy to the urgency of the situation

The Operational Performance results, shown in Fig. 6, indicate that the system’s conversational behavior varies with both the phase of the call and the urgency of the Chief Complaint. Analysis of information collection efficiency by call phase (Fig. 6a) reveals an inverted U-shaped trend, with efficiency logically peaking at an average score of 4.3 during the critical Assessment phase (simulated 2–5 minutes), when rapid, structured questioning is most important. Efficiency remains relatively high in the subsequent Guidance and Closure phases, suggesting that the system does not substantially slow down or become disorganized once a preliminary assessment has been formed.

When examining performance over the call’s duration (Fig. 6b), the system also appears to prioritize urgent cases. The collection completeness for Life-Critical Events (green line) increases more rapidly and plateaus earlier than for traumatic or individual complaints, indicating an accelerated information-gathering strategy when stakes are highest. Importantly, the completeness curves for the three groups converge by the end of the call, suggesting that faster early questioning for life-critical events does not come at the expense of overall information coverage.

This prioritization is most evident in the system’s simulated response time (Fig. 6c). The agent is faster when handling Life-Critical Events (average 1.8 s per dispatcher turn) compared with Traumatic Incidents (2.1 s) and Individual Complaints (2.4 s), reflecting a shift toward shorter, more direct, and more instructional utterances once a life-threatening scenario is recognized. Analysis of total simulated call duration shows a similar pattern, with life-critical events having the shortest median duration (Supplementary Fig. 1). Taken together, these results suggest that, within our simulated environment, the system adapts its conversational pacing to the urgency of the situation while still achieving comparable end-of-call information completeness across complaint types.

Importantly, shorter simulated duration or faster per-turn response time should not be interpreted as inherently “more effective” call handling. In real EMS calls, interactional trouble (e.g., misunderstanding, resistance, role/knowledge mismatches) can require repair sequences, clarification, and repeated confirmation that appropriately lengthen the exchange but improve safety and shared understanding. Our timing analyses therefore reflect efficiency under controlled simulation assumptions and should be interpreted alongside interactional alignment outcomes rather than as a standalone proxy for call quality.

## Discussion

In this study, we developed and evaluated a novel multi-agent system for simulating emergency medical dispatch, grounded in clinical taxonomy and powered by LLMs. Our hybrid evaluation, combining expert physician assessment with automated linguistic metrics, demonstrated that the system can generate clinically plausible dialogues that adhere to established protocols under controlled conditions. The agent-based dispatcher achieved high ratings for the effectiveness of its operational actions, efficacy of its pre-arrival guidance, and interactional alignment. These findings suggest that grounding LLM agents in a structured, domain-specific knowledge framework can improve the safety and consistency of model behaviour in simulation, and may provide a useful foundation for the design of future decision-support tools in high-stakes medical environments.

Although our evaluation focuses on simulated conversations rather than patient-level outcomes, the dimensions we measure are mechanistically linked to clinical and operational endpoints in prior work. Higher **Guidance Efficacy**—for example, timely, clear pre-arrival instructions about CPR, positioning, or monitoring—is associated with increased bystander action and improved survival in time-critical emergencies. Similarly, better **Dispatch Effectiveness**, including earlier and more reliable location acquisition, targeted questioning to elicit red-flag symptoms, and correct involvement of additional services (such as police, fire, or poison control), is closely tied to shorter response times, more appropriate resource allocation, and reduced risk of missed or mis-prioritised cases. Strong **Interactional Alignment**—such as explaining why questions are necessary, managing resistance without escalation, and achieving a clear shared framing of the main problem—supports caller cooperation and adherence to instructions, which can indirectly affect both safety and efficiency. In the present study, we examine these mechanisms only in simulation under controlled conditions; any claim that improvements in these dimensions translate into better patient outcomes or operational performance would require prospective validation on real emergency calls and comparison against existing dispatch systems.

Consistent with prior communication research, the controlled framing ablation indicates that interaction quality is sensitive to how protocol actions are linguistically realized. In particular, single-focus sequential questioning appears to reduce caller cognitive load and minimize repair, supporting higher information completeness and stronger shared framing, while bundled or overloaded question turns increase interactional friction and reduce perceived timeliness. These findings support the use of the platform as a sandbox for systematically comparing protocol phrasing variants under standardized conditions, while avoiding overinterpretation beyond simulation (Supplementary Methods S6).

Our results highlight the likely importance of the taxonomy and fact commons in steering agent behaviour. The system’s ability to dynamically classify Chief Complaints and retrieve corresponding protocols at each conversational turn appeared to help keep the dispatcher agent’s responses both fluent and procedurally appropriate in our test scenarios. This approach functionally mirrors the core principle of retrieval-augmented generation, where a generative model’s output is anchored to a reliable external knowledge source. By curating a detailed fact commons for each Chief Complaint, we provided the LLM with a structured “source of truth,” which appears to improve the predictability and clinical coherence of its generated content in our test scenarios and establishes a strong foundation for future work incorporating real-time RAG from validated medical databases.

Furthermore, the multi-agent architecture provides significant flexibility for creating realistic and adaptable EMS workflows. This framework moves beyond simple question–answering and simulates the dynamic, multi-party coordination inherent in emergency response. Beyond procedural progression, the trigger perturbation experiment demonstrates that the simulation can elicit and quantify interactional regimes that require repair work. Introducing caller resistance, role mismatch, or shared-framing challenges selectively altered interactional alignment and operational pacing while leaving several core procedural markers comparatively stable, consistent with the notion that EMS call quality depends on both protocol content and the interactional work required to sustain cooperation. These results support a more precise characterization of the framework as a controlled environment for studying misalignment and repair dynamics under standardized conditions (Supplementary Methods S8). The system’s modularity allows individual components to be independently updated or replaced, such as the caller agent’s persona, the dispatcher agent’s reasoning model, or the specific protocols used. This makes it possible to scale the simulation by adding new agents representing other emergency services (e.g., police, fire, poison control) or by integrating external tools and APIs [7], thereby creating a high-fidelity digital twin–like representation of dispatcher–caller workflows for research and design purposes. Within the confines of simulation, such a platform can be used to explore protocol variants, test operational hypotheses, and study communication dynamics before any consideration of clinical deployment.

At the same time, the LLM and MAS components of our system inherit well-known limitations of contemporary AI. Even when grounded in a fact commons, LLMs can hallucinate, overstate their certainty, or behave unpredictably when confronted with out-of-distribution inputs, while multi-agent systems can amplify coordination failures or subtle mis-specifications of roles and goals. Mitigating these risks will require a combination of technical and organisational safeguards. On the technical side, future extensions of this work could incorporate explainable AI techniques (for example, turn-level rationales, explicit links from each instruction to taxonomy rules, and visualisations of agent state) to make agent behaviour more transparent to clinicians and dispatch leaders. Robust data governance will also be essential: training and evaluation data, as well as simulation logs, will need to be managed under explicit privacy, security, and access controls that are compatible with EMS regulatory frameworks. On the organisational side, any future decision-support system derived from this architecture should be designed around strong human oversight, positioning the AI as a recommendation layer that dispatchers and physicians can interrogate, override, or ignore, and embedding mechanisms for uncertainty-aware behaviour (e.g., deferring to human-only workflows when model confidence is low or when scenarios fall outside the validated scope). Ultimately, any move beyond simulation would have to be contingent on rigorous real-world validation, prospective trials, benchmarking against existing systems, and formal regulatory evaluation, rather than assumed as an inevitable trajectory.

This inherent flexibility is particularly valuable for addressing the crucial need for social and cultural customization in emergency services. The current framework could, in principle, be adapted to better serve diverse communities. For instance, the LLM’s multilingual capabilities could be used to simulate interactions with non-native speakers, and the caller identity framework can be expanded to include personas representing various age groups, cognitive abilities, or cultural backgrounds that influence how symptoms are described and how instructions are received. By loading the fact commons with region-specific protocols or public health priorities, the simulation environment can support jurisdiction-specific scenario libraries and protocol tuning and, in future work, could also be repurposed as a resource for communication training. This aligns with broader reviews on the role of LLMs in crisis management and emergency medicine [62], while underscoring that responsible deployment will depend on aligning technical advances with governance, regulation, and frontline practitioner input.

### Limitations and future work

Despite the promising results, this study has several limitations. First, our evaluation was conducted in a simulated environment, which cannot fully replicate the high-stress, unpredictable nature of real-world 911 calls, including background noise and extreme caller emotional states, as well as low-affect/stoic presentations where urgency may be less overtly displayed in talk [60, 61]. While our main evaluation uses English dialogues, we additionally conducted an English-only stress test in which the caller agent’s functional English communication was constrained (fluent English vs LEP-English prompt setting). This supplementary analysis found no statistically significant between-condition differences across rubric dimensions, although guidance delivery showed the largest non-significant trends (Supplementary Methods S7). In this supplementary experiment, “limited proficiency” was operationalized as an English-only prompt constraint rather than true multilingual or interpreter-mediated communication. Specifically, the caller agent was instructed to remain English-speaking but use short phrases, simple vocabulary, grammatical errors, occasional misunderstanding, and greater difficulty responding to long or bundled questions. This manipulation was intended to approximate reduced functional English communication under stress, allowing a controlled test of how constrained comprehension and expression may affect dispatch interaction quality.However, this design does not capture true multilingual or code-switched interactions (e.g., mixed-language calls), nor does it evaluate how language identification, translation, or culturally specific phrasing might affect comprehension and repair; extending the framework to non-English and mixed-language dispatch scenarios remains an important direction for future work. Second, to simplify scenario generation and enable controlled comparisons, we fixed the caller location to a single placeholder address (“123 Main Street”) across cases. This choice reduces diversity in address formats and removes realistic complexities that often drive dispatch delays and miscommunication in practice (e.g., incomplete addresses, landmarks, apartment/building identifiers, rural locations, caller uncertainty, or mobile callers in transit). Future studies should introduce location variability and structured address perturbations to more faithfully test location elicitation, confirmation strategies, and error recovery under realistic conditions. Third, while our panel of four physicians provided substantial agreement, a larger and more diverse group of evaluators, including experienced paramedics and professional dispatchers, would provide a more holistic assessment of the system’s operational utility. The statistically significant variability observed between two raters on specific metrics highlights the importance of incorporating diverse professional perspectives. Furthermore, to guide iterative refinement, we conducted a qualitative root cause analysis of representative failure cases (detailed in Supplementary Method S5), identifying patterns such as “question overload” and “premature misclassification” (Supplementary Fig. 6 and Supplementary Table S11). Fourth, our taxonomy, while broad, is static; a real-world system would need mechanisms to continuously update its knowledge base with the latest clinical guidelines. Fifth, although we implemented several hallucination mitigation strategies—including grounding in a shared clinical knowledge base, strict prompting constraints, and scenario exclusion rules—we did not quantitatively measure hallucination rates or compare the constrained system against unconstrained LLM baselines. As a result, our claims about reduced hallucination risk are based on qualitative review and expert judgment in a sandboxed setting, and future work should include formal, quantitative evaluation of hallucination frequency and impact in both simulated and real-world data.

Future work will focus on addressing these limitations. Emerging evidence in emergency medicine also highlights the potential of AI-driven approaches [63]. A next step is to conduct prospective validation in controlled settings with professional dispatchers and paramedics, using our system as a simulation and analysis platform to compare protocol variants, examine information-gathering strategies, and test decision-support interface designs. We also plan to benchmark our system against existing models (e.g., EMS-BERT [64]) and standardized datasets to quantitatively measure its performance. Further development will focus on integrating a true RAG architecture to allow the agents to pull information from live, trusted medical sources. Finally, exploring the complex ethical considerations surrounding AI in emergency medicine—particularly safety, accountability, and the mitigation of algorithmic bias—will be a central theme of our ongoing research. Finally, we will expand the “Lack of Information” fallback from a safety constraint into an explicit object of study. In real calls, incomplete or unclear information is often resolved through conversational repair and incremental meaning-making (e.g., targeted clarification, rephrasing, confirmation checks, and landmark-based grounding). Future iterations will therefore model and evaluate structured fallback strategies—when to defer, what minimal clarifications to request, and how to confirm understanding—so that the framework can more faithfully capture how shared meaning is established under uncertainty rather than treating missing information solely as an error condition.

## Conclusion

In conclusion, this study shows that combining a structured clinical taxonomy with a multi-agent LLM framework can enable the simulation of complex EMS dispatch scenarios in a clinically grounded and operationally realistic manner under controlled conditions. The proposed approach provides a potential blueprint for developing and customising AI agents to support high-stakes medical communication, conditional on rigorous real-world validation. Rather than presupposing deployment, our findings point to a possible pathway for future training and decision-support tools that would operate under strong human oversight, robust data governance, and formal regulatory review. While further work in live dispatch environments is essential to assess impact, safety, and generalisability, this simulation-based framework establishes a foundation for advancing AI-assisted emergency medicine in both research and design.

## Electronic supplementary material

Below is the link to the electronic supplementary material.

Supplementary Material 1

## Data Availability

The underlying clinical data used in this study are from the MIMIC-III database. We cannot release the full clinical-text–derived datasets openly. Upstream licenses for MIMIC/PhysioNet credentialed data prohibit onward redistribution and the use of third-party online services; derivatives that could enable re-identification must remain under controlled access. Although de-identified, narrative notes still carry non-zero re-identification risk, which is why access is managed rather than public. Researchers interested in accessing the MIMIC-III data can do so via the standard PhysioNet credentialing and training process: https://physionet.org/. The online version contains supplementary material available at 10.5281/zenodo0.17221919.

## Abbreviations

EMS: Emergency Medical Services
EMD: Emergency Medical Dispatch
MAS: Multi-Agent System
LLM: Large Language Model
CC: Chief Complaint
RAG: Retrieval-Augmented Generation
MIMIC-III: Medical Information Mart for Intensive Care III
CPR: Cardiopulmonary Resuscitation
AC1: Gwet’s Agreement Coefficient 1
F: F-statistic (ANOVA)
*χ*^2^: Chi-squared statistic
LEP: Limited English Proficiency
HRSA: Health Resources and Services Administration
NHTSA: National Highway Traffic Safety Administration
MPDS: Medical Priority Dispatch System
EMDPRS: Emergency Medical Dispatch Priority Reference System
API: Application Programming Interface
NLP: Natural Language Processing
Flesch: Flesch Reading Ease score
