# Supplementary material for: DispatchMAS: fusing taxonomy and artificial intelligence agents for emergency medical services
Source: BMC Emerg Med. 2026 Mar 18;26:78. doi: 10.1186/s12873-026-01540-9 (PMC13001355; doi:10.1186/s12873-026-01540-9)
Supplement: Supplementary file 1 — Supplementary Material 1 [file 12873_2026_1540_MOESM1_ESM.docx]

# Supplementary Information

**Glossary of Technical Terms**

**Artificial intelligence (AI)**
Computer systems that perform tasks that usually require human intelligence, such as understanding language, recognizing patterns, or making decisions.

**Large language model (LLM)**
A type of AI system trained on large amounts of text so it can generate and understand human language. In this paper, the dispatcher and caller “agents” are implemented as LLMs.

**Multi-agent LLM system / multi-agent system (MAS)**
A setup where several AI “agents” (here, a caller agent and a dispatcher agent) interact with each other, each playing a different role in the simulation.

**Agent / agentic system**
An “agent” is a software component that can receive inputs, decide what to do, and generate outputs in an ongoing interaction. An “agentic system” is built from such agents acting in coordinated ways—in our case, to simulate emergency calls.

**Human-in-the-loop framework**
A design where humans remain actively involved in using, monitoring, or correcting an AI system. For example, clinicians reviewing simulated calls and giving feedback before any real-world deployment.

**Retrieval-augmented generation (RAG)**
A method where an LLM first “retrieves” relevant information from a knowledge source (e.g., guidelines), and then uses that information to generate its response. This helps keep outputs closer to established protocols.

**EMS dispatch taxonomy**
A structured set of categories and labels that describe emergency call types, chief complaints, and key dispatch decisions. It acts as a “map” of how calls are classified and handled in the system.

**Fact commons / shared clinical knowledge base**
A centralized, shared repository of clinically approved information (e.g., protocols, pre-arrival instructions, dispatch rules) that all AI agents can access. It is used to “ground” the agents so their responses follow the same medical facts and procedures.

**Clinical knowledge base**
More generally, an organized collection of clinical rules, guidelines, and reference content that an AI system uses to support consistent, evidence-informed behavior.

**Caller agent**
An AI agent that plays the role of the caller, describing symptoms, answering questions, and responding to instructions, based on a scenario description.

**Dispatcher agent**
An AI agent that plays the role of the emergency dispatcher, asking questions, identifying the main problem, locating the caller, and providing pre-arrival instructions in line with dispatch protocols.

**AutoGen framework**
A software framework for managing conversations between multiple AI agents. In this study, it coordinates the back-and-forth dialogue between the caller and dispatcher agents.

**Hallucination (LLM hallucination)**
When an LLM produces information that sounds plausible but is factually wrong or not supported by its knowledge base. Grounding the system in the taxonomy and shared clinical knowledge base is intended to reduce this risk.

**Sentiment analysis**
An automated method for classifying text as having a generally positive, negative, or neutral emotional tone. Here it is applied to dispatcher utterances to monitor overall emotional balance.

**Emotion classification**
An automated method that assigns more specific emotions (e.g., “calm,” “anxious,” “sad”) to text. In this work, it helps assess whether dispatcher responses remain emotionally appropriate during simulated crises.

**Clarity (readability)**
In the paper, “clarity” refers to how easy it is for an average person to understand the dispatcher’s words. We quantify this using a standard readability score (Flesch Reading Ease), where higher scores mean simpler, easier-to-follow language.

**Professionalism (politeness)**
An automated measure of whether a message is respectful, considerate, and non-abrasive. The underlying “politeness classifier” scores whether the dispatcher’s phrasing aligns with professional, patient-centered communication standards.

**Communication-quality metrics**
A set of automated measures (sentiment, emotion, clarity/readability, professionalism/politeness) used to assess how well the dispatcher communicates, beyond purely clinical correctness.

**Hybrid human–algorithm evaluation framework**
An evaluation approach that combines expert human ratings (e.g., physicians judging clinical appropriateness) with automated metrics (e.g., sentiment, clarity, professionalism) to form a more complete picture of system performance.

**Grounding (knowledge-grounded responses)**
Constraining the AI agents so that their outputs must be consistent with a specified set of facts or protocols (the taxonomy and shared clinical knowledge base), rather than relying solely on free-form text generation.

**Digital twin–like simulation environment**
A computer simulation that closely mimics real dispatcher–caller interactions, using realistic workflows and protocols. “Digital twin–like” here means it approximates real operations but is still a research/simulation environment, not a live operational system.

**Out-of-distribution (OOD) scenarios**
Cases or inputs that differ from what the system has seen during development (for example, atypical complaints or unusual combinations of symptoms). These are important for testing robustness.

**Prompt / prompt engineering**
The specific instructions, context, and examples given to an LLM to guide its behavior. Carefully worded prompts help the caller and dispatcher agents follow the desired protocol and style.

**Scenario template / standardized scenario**
A predefined structure describing the clinical situation (e.g., age, symptoms, setting, underlying condition) used to generate consistent, repeatable call simulations for evaluation.

# Supplementary Methods

**Supplementary Method S1. Taxonomy and Fact Commons: Sourcing and Fields**

**Purpose:** This study constructed a clinical taxonomy of 32 chief complaints (CCs) and a corresponding fact commons, grounded in established Emergency Medical Services (EMS) and Emergency Medical Dispatch (EMD) training and operational standards. This knowledge foundation serves to standardize the conversational context, provide the basis for pre-arrival instructions, and establish structured retrieval anchors for the system’s workflow.

**Sources and Curation:** The selection and definition of the 32 CCs were primarily informed by national and state-level EMS training curricula and dispatch protocols, encompassing adult, pediatric, and maternal entries. Terminology and key points were cross-referenced with established textbooks and public guidelines. To ensure adaptability across diverse scenarios, we adopted a curation principle of prioritizing standardized entries from official protocols. Where discrepancies in terminology or procedural order existed between sources, preference was given to expressions with a clearly defined scope of application and evidence grading, while specifying contextual limitations (e.g., industrial vs. residential exposure, single vs. multiple victims) within the relevant fields.

The taxonomy encompasses 32 CCs organized into three broad categories:

1. **Traumatic incidents (n = 11):** Animal Bites；Assault / Sexual Assault；Burns；Eye Problems / Injuries；Fall Victim；Heat / Cold Exposure；Bleeding；Industrial Accidents；Stabbing / Gunshot Victim；Traumatic Injuries；Vehicle-related Injuries.
2. **Individual CCs (n = 14):** Abdominal Pain；Allergies / Stings；Back Pain；Breathing Problems；Chest Pain；Convulsion / Seizure；Diabetic Problems；Headache；Heart Problems；Ingestions / Poisons / Overdose；Psychiatric / Behavioral；Sick Person；Stroke / CVA；Unknown Problem / Man Down.
3. **Time/life-critical events (n = 7):** CO / Inhalation / HAZMAT；Cardiac Arrest；Choking；Drowning；Electrocution；Pregnancy / Childbirth；Unconscious / Fainting.

**Field Schema (per CC):** Each CC is structured with a uniform field schema:

1. **Background & Key Identifiers:** Core pathophysiology and diagnostic clues, including common differential scenarios.
2. **Common Causes / Scene Patterns:**Typical etiologies and common caller-described scene patterns.
3. **Red-Flag Cues:** Warning signs requiring priority clarification or immediate intervention (e.g., critical items related to Airway, Breathing, Circulation).
4. **Pre-arrival Instructions:** Sequentially ordered, actionable guidance (one step at a time), including safety and self-protection reminders.
5. **Special Populations:** Specific considerations and differing thresholds for pediatric, maternal, geriatric, or comorbid populations.
6. **Referenced Tools / Protocols:** Associated assessment criteria or standardized procedures (e.g., priority reference systems, burn area estimation charts).
7. **Potential External Coordination:** External agencies that may be required for specific scenarios (e.g., Fire, HazMat, Poison Control), annotated with text descriptions of their triggering conditions.

**Worked Example (CO Exposure / Inhalational Hazards)**

1. **Background:** Carbon monoxide is a colorless, odorless gas that can cause headache, nausea, and altered consciousness; it is common in settings with faulty furnaces/generators and poor ventilation. Industrial leaks are a separate consideration for hazardous material inhalation.
2. **Scene Patterns:** Multiple victims simultaneously unwell, enclosed spaces, history of using combustion appliances. Group exposures may be seen in public venues or industrial parks.
3. **Red-Flags:** Altered consciousness/seizures, respiratory distress, cyanosis; ongoing exposure or inability to evacuate from the contaminated source.
4. **Pre-arrival (in order):** If safe to do so, evacuate the contaminated environment immediately. Avoid entry without proper protection. Ventilate the area as quickly as possible. If available and trained, administer high-flow oxygen. Continuously monitor consciousness and breathing. Call 9-1-1 again if symptoms worsen or new ones appear.
5. **Special Populations:** Children are more susceptible to CO due to higher metabolic rates and have lower toxicity thresholds. Pregnant women and their fetuses are at higher risk of hypoxia, warranting more aggressive transport and monitoring recommendations.
6. **Potential Coordination:** Fire Department/HazMat teams; Poison Control hotline (dependent on jurisdictional resources).

**Supplementary Method S2. Caller Agent Identity Generation and Calibration**

**Purpose:** This section delineates the design and calibration of the Caller Agent's identity classes and linguistic styles. The primary objective is to ensure that simulated interactions closely approximate the authentic context of EMD while systematically avoiding illogical or unsafe role-playing scenarios.

**Identity Classes (6):** Six distinct caller archetypes were defined to cover a comprehensive range of real-world scenarios:

1. **Patient (Self-reporting):** The individual directly experiencing the medical emergency.
2. **Family/Relative:** A family member or close friend of the patient, often with emotional involvement and some knowledge of the patient's history.
3. **Bystander/Neighbor:** An uninvolved third party who witnessed the event.
4. **Multiple Callers:** multiple concurrent callers, potentially providing conflicting information.
5. **Alleged Perpetrator / Involved Party:** An individual potentially responsible for the incident, a scenario governed by safety-first protocols.
6. **Limited-Proficiency Speaker:** A caller with linguistic barriers, such as a non-native speaker or an individual with speech impairments.

**Sourcing and Rationale:** Caller identity archetypes and their linguistic tendencies were grounded in clinical narratives from the MIMIC-III electronic health record dataset; utterance style was calibrated with linguistic samples from five real 9-1-1 transcripts to reflect the ambiguity, urgency, and emotional tone of actual emergency calls while avoiding overly precise or implausibly clinical language.

**Construction Workflow:** For each scenario, a Caller Agent is instantiated following a template and sampling from a predefined set of variables (see Table S7). This process is governed by three core principles:

1. **Contextual Coherence:** The identity must be consistent with the CC and the scene (e.g., a "Coworker" identity for an industrial accident).
2. **Layperson Plausibility:** The language and information provided by the agent must be reasonably attainable by an ordinary person in that situation.
3. **Safety-First Protocol:** The simulation avoids prompting dangerous actions or eliciting details that constitute self-incrimination, prioritizing scene safety.

**Sampling Variables:** Key variables sampled to construct each unique caller persona include: relationship to the patient, scene/location, time of day, emotional tenor (e.g., calm, worried, anxious, panicked), degree of uncertainty (what is known vs. unknown), language proficiency, and observable, non-clinical facts.

**Style Calibration:** The agent's utterances are designed to be short and colloquial, incorporating natural speech patterns like fillers ("um...") and self-corrections ("I think... no, wait..."). The agent is explicitly prohibited from generating professional diagnoses, medication names, or lab values. Factual statements are bounded by firsthand or secondhand knowledge (e.g., "I see," "I heard," "I was told").

**Safety and Logical Consistency Rules:** A set of hard-coded rules ensures the safety and plausibility of every simulation:

1. A patient who is unconscious, seizing, or otherwise incapacitated cannot be assigned the role of the caller.
2. Non-relatives (e.g., bystanders) do not provide precise age or detailed medical history unless this information was explicitly communicated to them at the scene.
3. In scenarios involving an alleged perpetrator, the Dispatcher Agent prioritizes securing the location and assessing immediate safety risks over pursuing details that constitute self-incrimination.
4. The Caller Agent is never prompted to perform or suggest unsafe actions (e.g., entering a hazardous environment, moving a patient with a potential spinal injury).
5. Linkage to CC / Scene. Caller identities are probabilistically linked to the scenario context. For example, a syncope event in a public park is more likely to be reported by a bystander, whereas a CC at a residence is more likely to be reported by a family member.

**Language and Accessibility:** The "Limited-Proficiency Speaker" identity uses simpler sentence structures and may express confusion or request repetition to simulate realistic communication challenges. The agent avoids idioms and complex syntax.

**Quality Control:** Prior to the main evaluation, a caliber calibration was performed on 10 pilot cases. Two researchers independently reviewed each simulated dialogue against the rules above to check for plausibility and consistency. Discrepancies were resolved through discussion to achieve consensus, and the feedback was used to refine the generation templates.

**Exclusion Criteria:** Any generated scenario is automatically excluded and regenerated if the caller identity:

1. Contradicts the factual basis of the scene.
2. Possesses unreasonable "privileged knowledge" (e.g., a bystander knowing a complex medical history).
3. Involves or suggests an unsafe action.
4. Discloses unnecessary or overly sensitive information.

**Supplementary Method S3. Multi-Agent System and Turn-Level RAG**

We implement a two-agent simulation, Caller and Dispatcher, within an AutoGen-based framework. Scenarios are seeded by EHR-derived narratives (MIMIC-III) to select from six realistic caller identities; the conversation is guided by a 32 CCs taxonomy and a six-phase call-handling protocol. At every turn, the Dispatcher applies turn-level CC classification with a safety fallback (“Lack of Information”) and then performs RAG to inject CC-specific procedural knowledge and pre-arrival instructions from the fact commons into its next utterance. When protocols indicate, the system can escalate to auxiliary resources (e.g., EMDPRS, police, fire) via function-like calls; in this study, auxiliary responses were mocked to maintain flow. Calls conclude with a standardized safety-net closure (e.g., instructing the caller to call back if anything changes).

**Turn-level loop (t):**

1. Classify CC from dialogue so far → one of 32 CCs, else Lack of Information.
2. Retrieve CC-bound protocol elements (red-flags, steps, pre-arrival guidance).
3. Condition the Dispatcher’s prompt with retrieved facts; generate one focused next move (a question or an instruction) in plain language.
4. If indicated, invoke auxiliary resource (mocked in this study) and integrate the result.
5. Deliver pre-arrival instructions when criteria are met; continue reassessment.
6. On dispatch confirmation / completion, provide safety-net and close the call.

Note. This module is executed within the same prompt discipline described in Method S4 (e.g., turn discipline and standardized closure), ensuring consistency and reproducibility.

**Supplementary Method S4. Prompt Library**

We developed a prompt library for the system's four core components: the dispatcher system prompt, the caller system prompt, the CC classifier prompt, and the turn-level RAG template. The full text of each prompt is provided in Tables S2–S5. This library is designed to standardize agent behavior, mitigate generative drift, and ensure replicability. Our design follows a set of global constraints and component-specific rules.

**Global Hard Constraints (Enforced Across All Prompts):**

1. **Single-Action Turns:** Agents are restricted to a single primary action per turn (e.g., one question or one instruction).
2. **Layperson Language:** All communication must use plain, non-technical language appropriate for a distressed lay caller.
3. **Safety-First Ordering:** Red-flag assessment and critical pre-arrival instructions must precede less urgent questioning.
4. **Standardized Closure:** Calls must conclude with a standardized closing that includes callback contingencies. Off-protocol advice and medical diagnoses are strictly prohibited.

**Component-Specific Notes:** The dispatcher prompt encodes turn discipline and the mandatory closing phrase. The caller prompt specifies the assigned role, enforces a colloquial tone, and establishes lay-knowledge boundaries to prevent the generation of medical jargon or overly sensitive details. The CC classifier prompt requires a deterministic JSON output, mandating a selection from the predefined taxonomy or the "Insufficient Information" label without any free-text rationale. Finally, the RAG template is structured to inject only turn-appropriate, factual content from the fact commons (e.g., red-flag cues, ordered instructions) and explicitly forbids creative expansion by the LLM. For traceability, each prompt includes minimal header metadata (e.g., version, date), with any revisions documented.

**Supplementary Method S5. Qualitative Analysis of Failure Cases**

To supplement the quantitative metrics, we conducted a qualitative root cause analysis on a representative set of failure cases, as illustrated in Supplementary Figure 6. This analysis was designed to identify specific error patterns and inform the iterative refinement of the system's operational logic. The process involved dissecting simulation logs where the system violated core protocols or received low performance ratings to trace error propagation from its origin.

As shown in Case #1 of the figure, one primary issue was the violation of the single-action-per-turn constraint. The dispatcher agent asked multiple questions at once, which led to caller confusion and delayed the acquisition of critical information. Case #2 demonstrates another significant failure pattern stemming from the premature misclassification of a CC. The system incorrectly identified "Abdominal Pain" as "Poisoning," which caused it to retrieve an incorrect protocol and provide inappropriate guidance, highlighting how classification accuracy is a critical bottleneck for the entire workflow .

Furthermore, Case #3 illustrates a critical timing failure in a time-sensitive emergency. The system failed to recognize the severity of a cardiac event promptly, prioritizing routine data collection over immediate life-saving instructions. This created a significant "Performance Gap" between the ideal and actual intervention quality, with potentially severe clinical repercussions . The findings from this qualitative analysis were used to define stricter operational constraints and methodological refinements, such as enforcing a priority-based mode-switching mechanism for critical cases and improving the agent's ability to handle ambiguity before committing to a CC classification.

# **Supplementary Method S6. Question Framing Experiment**

We ran question-framing experiments to make the platform’s purpose concrete: even when the clinical scenario is identical, the dispatcher’s wording can change how quickly callers understand, how much they disclose, and whether they cooperate. In turn, that affects near-term operational outcomes that plausibly matter for patients—getting location sooner, eliciting key symptoms reliably, triggering the right escalation/agents, and delivering pre-arrival guidance the caller can follow.

In Experiment 1, we held the underlying scenarios and evaluation rubric constant and varied only the dispatcher’s questioning style (implemented as style constraints in the dispatcher prompt). We then scored each call using the same taxonomy-based dimensions already used in the paper (guidance, dispatch effectiveness, alignment/trouble management, and operational dynamics), reporting both task completion rates (e.g., location obtained) and quality ratings (mean ± SD).

Interpreting the results, Single-Focus Sequential performs best overall and most consistently: it has the highest rate of advice given (90%), the best caller-facing ratings (satisfaction 4.00; helpfulness 4.11), and the strongest operational markers (correct other agents 98%; location obtained 95%; call-back instruction 72%). It also leads on the core mechanism the reviewer cares about—eliciting complete, usable information without breaking alignment—with the highest frame alignment (4.08), shared medical problem framing (4.05), stance/role alignment (4.12), information gathering completeness (4.15), and near-top response timeliness (3.98). This pattern supports the claim that reducing cognitive load and keeping a clear one-question/one-answer rhythm improves both cooperation and completeness.

By contrast, Overloaded Bundle is the weakest and illustrates the “what not to do” case the reviewer asked for. It shows lower satisfaction (3.59), lower helpfulness (3.66), fewer questions successfully asked/answered (3.52), weaker frame alignment (3.52), poorer completeness (3.65), and slower perceived timeliness (3.50). This is consistent with the idea that bundling multiple requests in one turn increases missed items and resistance, forcing repair and reducing overall efficiency.

The middle conditions show tradeoffs rather than uniform gains. Explicit Jargon maintains solid operational performance (e.g., location 92%, other agents 92%, guidance accuracy 4.03, completeness 4.00) but has lower caller experience metrics (satisfaction 3.82; alignment 3.85), suggesting jargon can preserve “clinical correctness” while subtly degrading mutual understanding. Medicalized leading/confirmatory slightly improves shared framing (3.85) and timeliness (3.85) but does not help key logistics (location 80%), consistent with a risk of narrowing the conversation toward a presumed diagnosis at the expense of foundational dispatch information. Overall, the experiment yields a clear, actionable contrast: single-focus sequential questioning is the most robust strategy, while bundled questioning is reliably harmful, and jargon/medicalized framing can introduce hidden alignment costs even when operational boxes are still checked.

| **Category** | **Sub-Category** | **Chunked Dual Explicit** | **Jargon Medicalized** | **Leading Confirmatory** | **Overloaded Bundle** | **Single Focus Sequential** |
| --- | --- | --- | --- | --- | --- | --- |
| Guidance  Efficacy | Advice given | 80% | 85% | 85% | 80% | 90% |
|  | Satisfaction with amount of advice | 3.75 ± 0.90 | 3.82 ± 0.92 | 3.74 ± 0.74 | 3.59 ± 0.70 | 4.00 ± 0.85 |
|  | Helpfulness of advice, if given | 3.88 ± 0.93 | 3.97 ± 0.79 | 3.79 ± 0.83 | 3.66 ± 0.81 | 4.11 ± 0.74 |
| Dispatch  Effectiveness | Number of questions asked and answered | 3.60 ± 0.77 | 3.80 ± 0.87 | 3.70 ± 1.08 | 3.52 ± 0.74 | 4.00 ± 0.71 |
|  | Relevance of questions asked and answered | 3.65 ± 0.88 | 3.88 ± 0.87 | 3.90 ± 0.83 | 3.70 ± 0.87 | 4.10 ± 0.83 |
|  | Contact the correct potential other agents | 82% | 92% | 90% | 88% | 98% |
|  | Told to call back if necessary | 58% | 68% | 70% | 58% | 72% |
|  | Location obtained | 80% | 92% | 80% | 82% | 95% |
| Interactional Alignment | Frame Alignment & Information Management | 3.62 ± 0.58 | 3.85 ± 0.79 | 3.83 ± 0.74 | 3.52 ± 0.81 | 4.08 ± 0.82 |
|  | Epistemic Appropriateness: Establishing caller’s knowledge basis | 70% | 72% | 80% | 75% | 85% |
|  | Epistemic Appropriateness: Shared medical problem framing | 3.65 ± 0.76 | 3.80 ± 0.90 | 3.85 ± 0.88 | 3.62 ± 0.66 | 4.05 ± 0.71 |
|  | Interactional Trouble Management: Handling resistance to questions / instructions | 3.62 ± 0.62 | 3.85 ± 0.79 | 3.73 ± 0.81 | 3.65 ± 0.82 | 4.00 ± 0.95 |
|  | Interactional Trouble Management: Alignment with caller’s stance and role | 3.83 ± 0.97 | 3.95 ± 0.97 | 3.92 ± 0.65 | 3.67 ± 0.91 | 4.12 ± 0.71 |
| Operational Performance Dynamics | Guidance accuracy | 3.88 ± 0.93 | 4.03 ± 0.72 | 3.73 ± 0.59 | 3.80 ± 0.93 | 4.05 ± 0.67 |
|  | Information gathering completeness | 3.83 ± 0.80 | 4.00 ± 0.87 | 4.00 ± 0.77 | 3.65 ± 1.01 | 4.15 ± 0.73 |
|  | Response timeliness | 3.58 ± 1.05 | 3.83 ± 0.83 | 3.85 ± 0.94 | 3.50 ± 1.00 | 3.98 ± 0.85 |

# **Supplementary Method S7. Language Proficiency Experiment**

We conducted an experiment to examine how caller language proficiency—a common source of real-world communication friction—affects call progress and outcomes within our simulation framework. Limited English proficiency (LEP) can increase clarification needs, reduce precision in symptom descriptions, and introduce repair sequences (e.g., “I don’t understand,” paraphrasing, or confirmation checks). These interactional pressures can plausibly influence both information elicitation (what the dispatcher successfully obtains) and guidance delivery (whether pre-arrival advice is provided clearly and acted upon), even when the underlying clinical scenario is unchanged. This experiment therefore tests whether the system’s measured performance is robust to linguistically constrained caller behavior, and where vulnerabilities may emerge.

To clarify the manipulation, all callers in this experiment were instructed to speak in English. Accordingly, the language proficiency factor did not represent language switching, non-English speech, or accent alone. Instead, it represented the caller’s functional ability to communicate in English during an emergency interaction. In the fluent English condition, the caller was instructed to speak naturally in complete sentences. In the LEP-English condition, the caller was instructed to remain English-speaking but communicate with short phrases, simple words, grammar errors, limited vocabulary, and occasional misunderstanding. Thus, the contrast was not whether the caller spoke English, but how effectively and flexibly the caller could understand and express information in English under stress.

At the prompt level, the LEP-English caller was further constrained to exhibit several specific behaviors intended to mimic limited functional English communication. These included using simplified utterances (e.g., telegraphic or fragmented sentence structure), avoiding complex vocabulary, showing greater difficulty with long or bundled questions, and responding more reliably to closed yes/no questions than to open-ended prompts. The caller was also instructed to signal comprehension difficulty explicitly (e.g., indicating confusion or asking for simpler wording) and to become clearer over time when the dispatcher used shorter, simpler, and better-structured questions. This design was intended to approximate a common real-world communication challenge in emergency dispatch: the caller can speak some English, but reduced comprehension and expressive precision increase the need for clarification, repetition, and repair.

We emphasize that this was a controlled prompt-based simulation manipulation, rather than a validated sociolinguistic or psycholinguistic assessment of English proficiency. The experiment therefore captures a narrow construct: constrained English communication performance within a standardized emergency-call scenario. It does not encompass the full heterogeneity of real-world limited English proficiency, such as bilingual code-switching, interpreter use, regional dialect variation, or culturally specific communication norms. In addition, we include representative example transcripts in Supplementary Table S12, allowing readers to directly inspect how these prompt-level language constraints were realized in the simulated calls.

Design-wise, we held scenarios, dispatcher behavior policy, and the taxonomy-based scoring rubric constant, and manipulated only the caller agent’s language proficiency constraint: a Fluent English condition and an LEP English condition. Each condition comprised 40 calls, and we evaluated the same outcome families as elsewhere in the paper—binary operational markers (reported as percentages) and graded rubric ratings (mean ± SD). We added inferential testing to quantify differences: Fisher’s exact tests for proportions and two-sided Welch’s t-tests for rating-scale outcomes; p-values are reported in the table.

Across all measures, performance was high in both conditions, and no between-condition differences reached statistical significance (all p > 0.05). The largest (still non-significant) differences appear in guidance delivery: advice was provided slightly less often in the LEP caller condition (98% vs 88%, p = 0.201), and guidance accuracy trended lower (4.30 ± 0.60 vs 4.12 ± 0.60, p = 0.184), with a similar direction for helpfulness of advice (4.10 ± 0.74 vs 3.94 ± 0.67, p = 0.314). In contrast, core dispatch effectiveness indicators were very similar, including question relevance (4.35 ± 0.69 vs 4.28 ± 0.55, p = 0.617) and location obtained (98% vs 95%, p = 1.000). Interactional alignment scores also showed small, non-significant differences (e.g., frame alignment 3.77 ± 0.65 vs 3.67 ± 0.72, p = 0.516; shared problem framing 3.85 ± 0.69 vs 3.75 ± 0.62, p = 0.497). Taken together, these results suggest that, within controlled simulations, LEP-like caller constraints do not substantially degrade overall task completion or alignment, while guidance delivery quality may be the most sensitive component and merits targeted mitigation (e.g., simpler instruction templates, explicit confirmation checks) in future protocol design experiments.

| **Category** | **Sub-Category** | **Fluent English** | **Lep English** | **p-value** |
| --- | --- | --- | --- | --- |
| Guidance Efficacy | Advice given | 98% | 88% | 0.201 |
|  | Satisfaction with amount of advice | 4.10 ± 0.63 | 4.11 ± 0.75 | 0.949 |
|  | Helpfulness of advice, if given | 4.10 ± 0.74 | 3.94 ± 0.67 | 0.314 |
| Dispatch Effectiveness | Number of questions asked and answered | 3.70 ± 0.81 | 3.80 ± 0.68 | 0.552 |
|  | Relevance of questions asked and answered | 4.35 ± 0.69 | 4.28 ± 0.55 | 0.617 |
|  | Contact the correct potential other agents | 90% | 92% | 1.000 |
|  | Told to call back if necessary | 28% | 20% | 0.600 |
|  | Location obtained | 98% | 95% | 1.000 |
| Interactional Alignment | Frame Alignment & Information Management | 3.77 ± 0.65 | 3.67 ± 0.72 | 0.516 |
|  | Epistemic Appropriateness: Establishing caller’s knowledge basis | 80% | 85% | 0.770 |
|  | Epistemic Appropriateness: Shared medical problem framing | 3.85 ± 0.69 | 3.75 ± 0.62 | 0.497 |
|  | Interactional Trouble Management: Handling resistance to questions / instructions | 3.95 ± 0.97 | 3.90 ± 0.73 | 0.795 |
|  | Interactional Trouble Management: Alignment with caller’s stance and role | 4.17 ± 0.74 | 4.17 ± 0.67 | 1.000 |
| Operational Performance Dynamics | Guidance accuracy | 4.30 ± 0.60 | 4.12 ± 0.60 | 0.184 |
|  | Information gathering completeness | 4.05 ± 0.59 | 4.15 ± 0.57 | 0.443 |
|  | Response timeliness | 4.08 ± 0.57 | 4.08 ± 0.75 | 1.000 |

# **Supplementary Method S8. Interactional Alignment Experiment**

To evaluate whether the simulation framework captures interactional misalignment and repair processes that commonly arise in EMS calls, we conducted a controlled trigger-based experiment in which caller behavior was systematically perturbed while holding the clinical scenario content, dispatcher policy, and scoring rubric constant. In real emergency interactions, breakdowns often stem less from the absence of protocol questions and more from conversational contingencies—caller resistance to questioning, uncertainty about the caller’s relationship to the patient, or an initially vague everyday account that requires negotiation toward a shared problem definition. These contingencies can introduce repair sequences (clarification, justification, reframing) that alter both the efficiency and completeness of dispatch communication.

We operationalized three empirically plausible “trouble sources”—Resistance, Role Mismatch, and Shared Framing—and compared them against a No Trigger baseline. Each condition consisted of 40 simulated calls. Outcomes followed the paper’s taxonomy-based rubric and included both binary operational markers (e.g., advice given, location obtained) and graded quality ratings (mean ± SD) spanning guidance efficacy, dispatch effectiveness, interactional alignment, and operational performance dynamics. We tested for omnibus differences across the four conditions using one-way ANOVA for rating-scale outcomes and chi-square tests for binary proportions; p-values are reported alongside the summary statistics.

Across conditions, core procedural performance remained relatively stable. Advice provision was consistently high (95–98%), and dispatch questioning performance showed minimal variation in both throughput (number of questions asked and answered) and perceived relevance. In contrast, measures explicitly tied to interactional coordination exhibited greater sensitivity to caller perturbations. Frame Alignment & Information Management showed a borderline overall difference across conditions (p = 0.054), with the lowest alignment under the Resistance condition (3.52 ± 0.77) relative to baseline (3.92 ± 0.61). The most pronounced effect was observed for Interactional Trouble Management—Handling resistance to questions/instructions, which differed significantly across conditions (p = 0.007) and dropped under Resistance (3.62 ± 0.80) compared with No Trigger (4.17 ± 0.63). These patterns indicate that the simulation is capable of eliciting and differentiating misalignment regimes that are not reducible to simple question–answer correctness, and that these regimes concentrate their impact on repair and alignment rather than on the dispatcher’s ability to continue asking protocol-relevant questions.

Operational performance dynamics further suggest that misalignment can carry time costs. Response timeliness differed significantly across conditions (p = 0.038), with the lowest timeliness under Resistance (3.80 ± 0.68) and higher timeliness in the No Trigger baseline (4.22 ± 0.69). Although several binary milestones (e.g., location obtained) showed numeric variation (e.g., 98% baseline vs 90% under Resistance), these differences were not statistically significant in this sample. Taken together, the results support a mechanistic interpretation: caller-induced trouble sources primarily tax the interactional work required to maintain cooperation and shared framing, which in turn is reflected in lower repair/handling scores and reduced perceived timeliness, while leaving much of the dispatcher’s protocol progression comparatively intact under controlled conditions.

| **Category** | **Sub-Category** | **No Trigger** | **Resistance** | **Role Mismatch** | **Shared Framing** | **Overall p-value** |
| --- | --- | --- | --- | --- | --- | --- |
| Guidance  Efficacy | Advice given | 95% | 95% | 95% | 98% | 0.930 |
|  | Satisfaction with amount of advice | 4.08 ± 0.48 | 4.08 ± 0.70 | 4.00 ± 0.65 | 4.03 ± 0.73 | 0.930 |
|  | Helpfulness of advice, if given | 4.05 ± 0.65 | 4.13 ± 0.73 | 4.05 ± 0.69 | 4.03 ± 0.53 | 0.907 |
| Dispatch  Effectiveness | Number of questions asked and answered | 3.80 ± 0.64 | 3.88 ± 0.71 | 3.77 ± 0.79 | 3.80 ± 0.51 | 0.898 |
|  | Relevance of questions asked and answered | 4.20 ± 0.51 | 4.10 ± 0.77 | 4.20 ± 0.64 | 4.17 ± 0.67 | 0.891 |
|  | Contact the correct potential other agents | 98% | 92% | 98% | 90% | 0.365 |
|  | Told to call back if necessary | 30% | 18% | 32% | 28% | 0.451 |
|  | Location obtained | 98% | 90% | 95% | 92% | 0.545 |
| Interactional  Alignment | Frame Alignment & Information Management | 3.92 ± 0.61 | 3.52 ± 0.77 | 3.73 ± 0.74 | 3.85 ± 0.61 | 0.054 |
|  | Epistemic Appropriateness: Establishing caller’s knowledge basis | 90% | 82% | 88% | 85% | 0.788 |
|  | Epistemic Appropriateness: Shared medical problem framing | 3.88 ± 0.68 | 3.88 ± 0.75 | 3.85 ± 0.73 | 3.95 ± 0.80 | 0.942 |
|  | Interactional Trouble Management: Handling resistance to questions / instructions | 4.17 ± 0.63 | 3.62 ± 0.80 | 3.92 ± 0.69 | 4.10 ± 0.89 | **0.007** |
|  | Interactional Trouble Management: Alignment with caller’s stance and role | 4.12 ± 0.56 | 4.17 ± 0.59 | 4.12 ± 0.60 | 4.15 ± 0.73 | 0.980 |
| Operational Performance Dynamics | Guidance accuracy | 4.22 ± 0.42 | 4.20 ± 0.68 | 4.25 ± 0.54 | 4.17 ± 0.59 | 0.935 |
|  | Information gathering completeness | 4.10 ± 0.80 | 4.25 ± 0.66 | 4.17 ± 0.67 | 4.15 ± 0.65 | 0.811 |
|  | Response timeliness | 4.22 ± 0.69 | 3.80 ± 0.68 | 3.92 ± 0.65 | 4.08 ± 0.72 | **0.038** |

# Supplementary Tables

**Supplementary Table S1. Taxonomy and Fact Commons Field Dictionary**

| **Field Name** | **Definition** | **Example (for "Chest Pain")** |
| --- | --- | --- |
| CC name | Standardized name of the chief complaint. | Chest pain |
| Complaint category | Broader taxonomy class for the CC. | Individual CC |
| Background & key identifiers | Clinical context and salient identifiers; include common look-alikes. | Possible cardiac origin; consider myocardial infarction; note exertional onset, radiation, diaphoresis. |
| Common causes / scene patterns | Typical etiologies or triggers; frequent scene patterns. | Myocardial infarction, anxiety, pulmonary embolism, musculoskeletal strain. |
| Caller-described symptoms / scene cues | How callers typically describe the symptoms or the scene. | Pressure or tightness; squeezing; pain radiating to arm/neck; shortness of breath; nausea. |
| Pre-arrival instructions (ordered) | Stepwise instructions the dispatcher may provide; safety first; scope-of-practice compliant. | Rest in a position of comfort (usually sitting up); loosen tight clothing; if prescribed and appropriate, take nitroglycerin; consider aspirin per local protocol; call back if symptoms worsen. |
| Special populations | Modifications for pediatric, maternal, geriatric, or other groups. | Children: cardiac etiology uncommon—consider asthma/trauma/anxiety; Pregnancy: consider PE/ACS, lower threshold for escalation. |
| Referenced tools / protocols | Relevant assessment tools or standardized protocols (do not expand here). | Priority reference system (cardiac) or local equivalent; 12-lead ECG trigger criteria (if available). |
| Potential external coordination (with triggers) | External agencies/resources that may need to be involved and the trigger conditions. | Advanced Life Support (ALS) unit if high-risk features; cardiology consult for suspected ACS; fire department for access issues. |

**Supplementary Table S2. Component and Versioning Baseline for Reproducibility**

| **Category** | **Component Name** | **Version / Checkpoint** | **Provider / Source** | **Role in System** |
| --- | --- | --- | --- | --- |
| **Core LLMs** | gpt-4-o | 0125 | OpenAI | Dispatcher & Caller Agents |
|  | gpt-4o | 0125 | OpenAI | CC Classification |
|  | gpt-4o | 0125 | OpenAI | Caller Identity & Background Generation |
| **Linguistic Analysis** | CardiffNLP/twitter-roberta-base-sentiment-latest | latest | Hugging Face / Cardiff NLP | Sentiment Analysis of Responses |
|  | j-hartmann/emotion-english-distilroberta-base | v1.0 | Hugging Face / j-hartmann | Emotion Recognition of Utterances |
|  | en_core_web_sm | 3.7.2 | spaCy | Text Preprocessing & Analysis |
| **Software Framework** | pyautogen | 0.2.16 | Microsoft / PyPI | Agent Conversation Orchestration |

**Supplementary Table S3. Full Prompt for Dispatcher Agent**

| **Section** | **Prompt Content** |
| --- | --- |
| Role & Objective | You are a dispatcher receiving a call from a caller who is reporting an emergency. Please try your best to help the caller and gather necessary information. Please speak in your role as a dispatcher, speak in a colloquial manner and give quick questions and instructions. Keep your response to 1 sentence or 1 question at a time. When appropriate, briefly explain why a question is needed, calmly manage caller resistance, and confirm a shared understanding of the main problem to support cooperation, safety, and efficiency. |
| Operational Protocol | **Emergency Call Handling Protocols** Please follow the Emergency Call Handling Protocols: 1. Gathering essential information when the call first comes in 2. Asking questions to understand the situation and environment at the scene 3. Sending the appropriate responders to the location based on the information gathered 4. Giving real-time updates to responders as new information comes in 5. Guiding callers with step-by-step actions to manage the situation until help arrives 6. Ending the call once the situation is resolved or the responders take over  7. Throughout, maintain interactional alignment (briefly justify key questions if the caller is confused or resistant; de-escalate; and summarize the main problem in plain language once clear). |
| Tone & Style Examples | **Tone** You should speak in a conversational manner and ask questions to gather information about the emergency. Keep your response short and quick, don't give long sentences and instructions. Here are some example sentences you can use, and you should follow a similar tone: - Ok, how old is this person having the stroke? - Ok, well who was talking to me earlier, telling me they were doing CPR? - Medical emergency operator 10. - Go back up to him and make sure he's laying flat on his back. (... and other stylistic examples from the code) |
| Hard Constraints & Prohibitions | **Notes** - Please speak in a colloquial manner, use easy-to-understand language and avoid using jargon. - NEVER ask several questions at once. Ask one question at a time and wait for the response. - NEVER give many instructions at once (No bullet point instructions). Give one instruction at a time and wait for the confirmation from the caller.  - If the caller resists or is distressed, acknowledge briefly and restate why you need the next question (“I know this is stressful—this helps me send the right help”).  - Once the situation is clear, provide a one-sentence plain-language summary of the main problem before continuing (e.g., “Okay—so she’s awake but breathing very fast”). |

**Supplementary Table S4. Full Prompt Template for Caller Agent**

| **Section** | **Prompt Content** |
| --- | --- |
| Role Definition | You are making a call to 911. Please play the role of a caller who is reporting the emergency. Always stay in your role. Please always speak in English. |
| Tone & Style Examples | **Tone** Please use conversational language and avoid using medical terms and long sentences. Based on the emergency, you might be anxious, worried, or scared. Please express your emotions in your speech. Here are a few example sentences of the caller's speech from other calls: - "Blood is coming out of my nose!" - "I'm not sure she's (pause) conscious yet, I'm outside." (... and other stylistic examples from the code) |
| Identity & Background (Dynamic) | **Identity and Background** Your Identity: patient / family / bystander / coworker / involved party / limited-proficiency speaker. Current Background: relation, location, time, observable non-clinical symptoms. |
| Operational Rules & Constraints | **Notes** 1. When asking for location, always reply "123 Main Street". 2. If you are not a family member, please do not give a detailed age of the patient, only give a general age range (e.g., 50-60). |

**Supplementary Table S5. Full Prompt for CC Classifier**

| **Section** | **Prompt Content** |
| --- | --- |
| Task Definition | Given an ongoing conversation, classify the scenario to one of the following incidents. The incident name you give should be in the given list of incidents. |
| Predefined Taxonomy (Dynamic) | Traumatic (11), Individual (14), Time/Life-Critical (7) (Note: This placeholder is dynamically populated with the full list of 32 CCs and their categories, sourced from the fact commons.) |
| Fallback Rule | If you cannot determine the incident type based on the given information, please classify it as "Lack of Information". |
| Output Format Constraint | Respond in JSON format: { "Incident Type": "<one of the 3 types or 'Lack of Information'>", "Incident Name": "<selected incident name>" } |

**Supplementary Table S6. Full Template for RAG Injection**

| **Component** | **Template Content** | **Hard Constraints & Prohibitions** |
| --- | --- | --- |
| Section Header | Other Agents/Tools | Injected content must appear under this header to separate it from the base prompt. |
| Knowledge Anchor | Manual for {incident_name}: {manual_str} | The {manual_str} must contain the formatted protocol for the classified {incident_name}. |
| Tool Availability | You are given this function call_another_agency... For this incident, you can call the following agents/tools: - "{agent_tool_str}" | The dispatcher is only permitted to call agents listed in {agent_tool_str}. |
| Post-call Behavior | ...Please take it as a reference and continue the conversation. Please keep your tone conversational... Do not given long sentences... | Must integrate the external information while maintaining the established conversational tone and constraints. |

**Supplementary Table S7. Caller Identity Template and Sampling Variables**

| **Variable** | **Allowed Values & Guidance** | **Illustrative Utterance** |
| --- | --- | --- |
| Caller Role (Class) | patient; family/relative; bystander/neighbor; coworker/on-scene adult; alleged perpetrator (safety-critical); limited-proficiency speaker | "I'm his wife, he's clutching his chest." |
| Relationship to Patient | self / spouse / parent / child / coworker / passerby / unknown | "I don't know him, we were at the bus stop." |
| Scene Context / Location | home / workplace / public place / roadway / industrial site / school / other | "In the underground parking lot, second floor." |
| Event Onset / Timeline | just now / minutes ago / hours ago / uncertain  (Guidance: Describes when the event started relative to the call.) | "It started about 10 minutes ago." |
| Emotional Tone | calm / worried / anxious / panicked (Guidance: Avoids extreme states that would render communication impossible.) | "I'm really starting to panic, he's not answering me." |
| Uncertainty Level | States unknown information explicitly; encouraged to use phrases like "I'm not sure." (Guidance: Reports only observable facts.) | "He's breathing, but I'm not sure if it's fast or slow." |
| Language Proficiency | native / non-native / limited speech (Guidance: Uses short sentences and may request repetition.) | "Sorry, can you say that again?" |
| Disallowed Content (Safety Rules) | Prohibited: Medical jargon, giving therapeutic advice, performing unsafe actions, providing self-incriminating details. | — |

**Supplementary Table S8. Models and Specifications for Automated Linguistic Analysis**

| **Analysis Component** | **Model Name** | **Architecture** | **Output Schema** |
| --- | --- | --- | --- |
| Sentiment Analysis | twitter-roberta-base-sentiment-latest | RoBERTa-base | {negative, neutral, positive} |
| Emotion Recognition | emotion-english-distilroberta-base | DistilRoBERTa-base | {joy, sadness, anger, fear, surprise, disgust, neutral} |
| Readability Analysis | Flesch Reading Ease | Statistical formula | Continuous score (0-100) |
| Core NLP Processing | en_core_web_sm | Transformer | POS, NER, DEP tags |

**Supplementary Table S9. Statistical Analysis of Inter-Rater Variability**

| **Metric** | **Test Used** | **Statistic** | **p-value** |
| --- | --- | --- | --- |
| **Guidance Efficacy** | | | |
| Satisfaction with amount of advice | ANOVA | F = 0.458 | 0.713 |
| Helpfulness of advice, if given | ANOVA | F= 1.367 | 0.259 |
| Advice given | Chi-squared | $\chi^{2}$=3.04 | 0.386 |
| **Dispatch Effectiveness** | | | |
| Number of questions asked | ANOVA | F = 2.264 | 0.088 |
| Relevance of questions asked | ANOVA | F = 4.301 | **0.007*** |
| Contact corrects other agents | Chi-squared | $\chi^{2}$=16.0 | **0.001*** |
| Told to call back if necessary | Chi-squared | $\chi^{2}$=6.77 | 0.342 |

***Statistically significant difference (p ≤ 0.05).**

**Supplementary Table S10. Scoring Methodology for Operational Performance Metrics**

| **Metric Name** | **Description & Calculation Method** | **Scoring Scale (1-5)** |
| --- | --- | --- |
| Information-Gathering Completeness | Measures the proportion of predefined critical information points (e.g., location, patient status) successfully elicited by the agent, relative to the total number of essential points defined for that specific emergency scenario. | 1: <60%  2: 60–79%  3: 80–89%  4: 90–95%  5: 96–100% |
| Response Timeliness | Measures the elapsed time from the start of the call to the first critical action, such as confirming dispatch or delivering a life-saving instruction. | 1: ≥9s or no critical action  2: 7-9s  3: 5-7s  4: 3-5s  5: <3s |
| Guidance Accuracy | Measures the adherence of the agent’s pre-arrival instructions to a gold-standard, protocol-specific checklist. The percentage of adherence is calculated by tokenizing and comparing the instructions. | 1: <70%  2: 70–84%  3: 85–92%  4: 93–97%  5: 98–100% |
| Overall Performance Score | A composite score calculated as a sum of the three metrics above, reflecting their relative clinical importance. | Sum: Guidance Accuracy Score + Information Completeness Score + Response Timeliness Score |

**Supplementary Table S11. Representative Failure Case Analysis**

| **Failure Type** | **Dialogue Snippet** | **Analysis** | **Suggested Improvement** |
| --- | --- | --- | --- |
| Question Overload | Dispatcher: "What's the address? How old is the patient? What are the symptoms? Are they conscious? Do they have any medical history?" Caller: "Uh... which question should I answer first?" | Violates the "one question at a time" constraint, confusing the distressed caller and delaying critical information gathering. | Adhere strictly to a sequential, prioritized questioning protocol: Location → Consciousness → CC → Key Symptoms. |
| Premature External Agent Call | Dispatcher: "Let me check the poison control guidelines." Caller: "No, he just has a stomachache from eating too much." | The agent prematurely classified "stomachache" as a "Poisoning" case based on insufficient information, leading to an unnecessary escalation. | The agent should be trained to ask clarifying questions (e.g., "Did you eat anything unusual?") before classifying a CC and triggering external agents. |
| Missed Safety-Net Instruction | Dispatcher: "The ambulance is on the way." Caller: "Should I call back if something changes?" Dispatcher: "TERMINATE THE CALL" | The agent failed to provide the critical safety-net instruction to call back if the patient's condition deteriorates, leaving the caller without guidance. | A hard-coded rule should ensure the phrase "If anything changes, call 9-1-1 again immediately" is delivered after confirming dispatch and before call termination. |
| Inappropriate Instruction | Dispatcher: "Try to give him some water to help with the chest pain." Caller: "He's having trouble swallowing." | Provided contraindicated advice (giving water) to a patient with potential cardiac chest pain, creating a risk of aspiration and delaying proper care. | The protocol should explicitly prohibit advising oral intake for suspected cardiac events. Instructions should focus on helping the patient find a position of comfort. |
| Use of Jargon / Unclear Language | Dispatcher: "Implement the recovery position protocol per EMDPRS guidelines section 4.2.1." Caller: "I don't understand what you mean." | The agent used technical jargon incomprehensible to a layperson, rendering the instruction ineffective and causing frustration. | Instructions must be translated from protocol language into simple, actionable steps. E.g., "Gently roll them onto their side. Then, tilt their head back a little to help them breathe easily." |
| Improper Prioritization in Critical Situations | Dispatcher: "What's your name and phone number?" Caller: "My husband is turning blue and not breathing!" Dispatcher: "I still need your contact information first." | The agent prioritized gathering administrative data over providing immediate life-saving instructions in a time-critical scenario (cardiac arrest). | The system's logic must prioritize critical interventions (e.g., CPR instructions) over standard data collection when life-threats like "not breathing" are identified. |

**Supplementary Table S11. Example Call Transcript Between Dispatcher and Caller**

| **Category** | **Information** |  |  |
| --- | --- | --- | --- |
| **Background** | It is a chilly Wednesday evening in early winter, around 7:30 PM, in a modest single-story home in a suburban neighborhood. The 51-year-old male patient, who has a known history of type II diabetes, a past stroke, and blood clot issues, lives there with his spouse. Over the past several months he has been in and out of rehab and the hospital, and recently returned home from a rehabilitation facility about a week ago after working on his strength and mobility.  The caller is his younger brother, who stopped by after work to check on him and bring dinner. When the brother arrived, he noticed the living room lights were on but the house was unusually quiet. The front door was unlocked. Walking into the living room, he found the patient sitting slumped sideways on the couch, still in a T-shirt and sweatpants, with the TV on but the remote on the floor. There were unopened takeout food containers on the coffee table and a half-finished glass of juice.  The patient appeared sweaty and pale, with clammy skin. His brother noticed that he seemed confused and slow to respond. When asked what day it was or what he had eaten, the patient gave jumbled or incorrect answers. His speech was somewhat slurred, but not as dramatically as during his previous stroke; he could form words, but he seemed disoriented. His eyes were open but he was staring off and needed repeated prompting to respond. He complained weakly that he was “just really tired” and “feeling weird.”  The brother knows the patient has diabetes and takes insulin and several other medications. On the end table by the couch, he sees the patient’s glucometer kit and insulin pens. The meter is out but not turned on, and there are test strips scattered around, suggesting the patient may have tried to check his blood sugar but didn’t complete it. When the brother asks if he has checked his sugar or taken his insulin, the patient can’t clearly remember and gives inconsistent answers.  Growing concerned, the brother helps him sit more upright and notices the patient seems weaker than usual and is having trouble keeping his head up. He’s breathing, but his breaths seem slightly deeper and more labored than normal. The brother feels that his skin is cold and damp. He doesn’t see any obvious injuries, no recent falls, and no signs of blood or trauma. The patient denies having chest pain but says his heart feels like it’s pounding. The brother, aware of his history of stroke and blood clots and worried about the possibility of a serious diabetic issue like very low or very high blood sugar, decides this is beyond something he can manage at home.  It’s already dark outside. The patient’s spouse is working a late shift and not at home, so the brother is alone with the patient. Realizing that his condition seems to be worsening and that he might not be safe to transport by car, the brother calls emergency services from his cell phone in the living room, reporting that his brother is a known diabetic who suddenly seems very confused, sweaty, and weak, and that he is worried he may be having a serious diabetic problem. | | |
| **Simulated Call Transcript** | [Dispatcher]: Hello, this is 911 dispatcher. What is your emergency?  [Caller]: Hi, uh—my brother, I’m at his house and he’s really out of it. He’s diabetic and he’s all sweaty and pale and confused and he’s not making sense. I think… I think something’s really wrong, maybe his blood sugar or a stroke or something. Can you please send someone?  [Dispatcher]: I’m sending help to you now—what’s the address where you are?  [Caller]: It’s, uh, 123 Main Street.  [Dispatcher]: Got it, 123 Main Street—what’s your brother’s age?  [Caller]: He’s 51.  [Dispatcher]: Okay, help is on the way—right now, is he awake and talking at all, or is he passed out?  [Caller]: He’s… he’s kind of awake, but he’s really out of it. His eyes are open, but he’s just staring. If I keep talking to him he’ll say a few words, but they’re all mixed up and slow. He’s not totally passed out, but he’s not really with it either.  [Dispatcher]: Okay, stay right with him—can you tell me if he’s sitting, standing, or lying down right now?  [Caller]: He’s on the couch, kind of slumped over, half sitting, half lying on his side. I propped him up a little but he keeps sort of… sliding down.  [Dispatcher]: Okay, I want him flat and safe—can you gently lay him on his side on the floor so he can’t fall, with his head supported?  [Caller]: Yeah, okay, hang on—… Alright, I got him. He’s on the floor now, on his side. I put a cushion under his head. He’s breathing, but it’s kinda heavy.  [Dispatcher]: Good job getting him down—does he have a glucometer there so you can check his blood sugar?  [Caller]: Yeah, he’s got it here on the table, the little meter thing with the strips and his insulin pens. I—I don’t know if he used it, there’s strips everywhere. Do you want me to try to check it? I can, I just… I’m not sure I’m doing it right.  [Dispatcher]: Yes, go ahead and check it—can you turn the meter on and tell me the number it shows after you do the finger poke?  [Dispatcher]: Okay, while you’re getting that ready, is his breathing steady, or is it getting slower or more shallow?  [Caller]: Uh, one sec, I’m trying to get this thing on—His breathing… it’s, uh, kind of heavy? Like deeper than normal. It’s not super fast, but it’s not stopping or anything. He’s still breathing, just… like he’s working at it a little.  [Dispatcher]: Okay, keep an eye on his breathing—did you get a blood sugar number on the meter yet, and what does it say?  [Dispatcher]: If the meter is still loading, that’s okay—tell me as soon as you see a number; right now, can he squeeze your hand if you ask him to?  [Caller]: Hang on—… Okay, I’ve got his hand. Hey, man, can you… can you squeeze my hand? … It’s like… it’s really weak. He kind of moved his fingers a little, but not a real squeeze. It’s barely anything.  [Dispatcher]: Okay, thank you—that’s helpful; now, look at his face and tell me, does one side of his mouth or face look droopy or uneven when he tries to talk?  [Caller]: Uh… hang on, let me look. Hey, hey, say something for me, okay? … It’s… I don’t know, maybe a little? His mouth looks kinda slack, but not like last time when it was really hanging. It’s more that he’s just sort of… not moving his face much. His words are slurred, but his face doesn’t look super crooked or anything.  [Dispatcher]: Okay, that’s fine—tell me, did you get a blood sugar number on the meter yet, and what does it show?  [Caller]: Yeah, yeah, sorry—uh, it finally came up. It says… 3–3–5. Three thirty-five. That’s bad, right?  [Dispatcher]: Okay, that is high but we can’t fix it at home—are his insulin pens right there, and has he taken any insulin in, say, the last hour, do you know?  [Dispatcher]: Okay, don’t give him any insulin right now—tell me, is he vomiting at all or saying his stomach hurts?  [Caller]: No, he’s not throwing up. I asked him if his stomach hurts and he just kind of shook his head a little. He hasn’t said anything about pain, just that he feels “weird” and really tired.  [Dispatcher]: Okay, good to know—put your ear close to his mouth and watch his chest for me: is he breathing at least once every 5 seconds?  [Caller]: Yeah, hang on—… Okay, I’m right next to him. One… two… three… four… Yeah, he took a breath. One… two… three… four… Another one. So it’s like every four seconds or so. He is breathing.  [Dispatcher]: Okay, keep him just like that on his side—does he have any chest pain, or does he grab at his chest at all?  [Caller]: No, he keeps telling me his chest doesn’t hurt. He said his heart feels like it’s pounding, but he’s not grabbing his chest or anything like that.  [Dispatcher]: Okay, that’s helpful—are the paramedics there yet, or do you still just hear sirens in the distance?  [Caller]: I don’t hear anything yet—no sirens, nothing outside. How far are they? Can you tell them to hurry, please? He looks worse, he’s just… he’s so out of it.  [Dispatcher]: They’re already on the way and I’ve marked this as urgent—I need to know, is he getting more sleepy now, like his eyes are closing and he’s harder to wake up?  [Caller]: Yeah… yeah, he is. He was kinda looking at me before, but now his eyes keep closing. Hey—hey, wake up, man… He’ll open them for a second if I call his name loud, but then they just sort of roll and close again. It’s definitely harder to get him to respond now.  [Dispatcher]: Okay, stay right by him—put one hand on his chest and one near his mouth, and tell me if his breathing suddenly gets slower, stops, or sounds very weird.  [Dispatcher]: You’re doing the right thing staying with him—right now, keep talking to him and tell me if you notice any change in his breathing or if he stops responding to you at all. | | |

**Supplementary Table S12. Example Call Transcript Between Dispatcher and Caller (Limited Language Proficiency Caller)**

| **Category** | **Information** |  |  |
| --- | --- | --- | --- |
| **Background** | It is a cool Sunday evening in early autumn, around 8:15 p.m., in a quiet suburban neighborhood. The patient, a 60-year-old married woman, is at home in her small two-story house. She is a practicing Protestant Quaker, generally calm and soft-spoken, and has a long, complicated cardiac history—including prior heart attacks, atrial fibrillation, a pacemaker, and diabetes. Earlier in the day she had attended a small meeting for worship and then had a light lunch. She mentioned to her friend that she felt a bit more tired than usual but assumed it was from not sleeping well due to her sleep apnea.  The caller, a close friend from her Quaker meeting, had come over in the late afternoon to drop off some homemade soup and stayed to chat. They are sitting together in the living room when the patient suddenly becomes quiet, placing her hand firmly on the center of her chest. She leans forward on the couch, her face tightening with discomfort. The friend notices that her color has changed—she looks unusually pale and a little sweaty around her forehead and upper lip. The patient says the discomfort started as a pressure in the middle of her chest about 20 minutes earlier, but she thought it would go away if she rested. Instead, it has gradually worsened and now feels like a heavy, squeezing pain that seems to spread slightly toward her left shoulder and up into her jaw.  The friend observes that the patient is slightly short of breath, speaking in shorter sentences, and seems more anxious than usual. The patient mentions she has had heart trouble in the past and a pacemaker, and that she’s worried this pain feels different from her usual indigestion or asthma symptoms. She denies any recent trauma or injury but says she feels a little nauseated and lightheaded. When she tries to stand to go to the kitchen, she has to sit back down, saying she feels too weak.  The friend has the patient sit upright on the couch with her feet on the floor. The patient’s breathing is a bit faster than normal, and the friend notices her pulse feels irregular and somewhat rapid when she reaches for the patient’s wrist, though she doesn’t know exact numbers. The friend asks if she has taken any nitroglycerin; the patient says she was prescribed it in the past but isn’t sure where the bottle is and admits she has not been good about keeping it close by.  It is quiet outside; the neighborhood is mostly residential with single-family homes. The friend realizes that because of the patient’s extensive heart history—prior myocardial infarction, valve surgery, pacemaker, atrial fibrillation, and diabetes—this chest pain could be very serious. The patient’s husband is out of town visiting one of their younger relatives, and there is no one else in the house. Deciding not to waste time, the friend uses her cellphone to call emergency services from the living room, giving the dispatcher the home address and explaining that they are with a 60-year-old woman with known heart problems who is having significant chest pain, shortness of breath, and sweating. While on the call, the friend stays beside the patient, trying to keep her calm and still, observing her breathing and responsiveness as they wait for paramedics to arrive. | | |
| **Simulated Call Transcript** | [Dispatcher]: Hello, this is 911 dispatcher. What is your emergency?  [Caller]: Uh, hello, yes—uh, my friend, she have bad chest pain.  She hold here—*chest*—she sweat, she breathe fast. I think… heart, maybe heart.  Please, you come fast, please.  [Dispatcher]: Okay, I’m sending help now.  What is the address where you are right now?  [Caller]: Uh, yes, address is 123 Main Street.  [Dispatcher]: Okay, I’ve got 123 Main Street, help is on the way.  How old is your friend?  [Caller]: She… uh… around maybe 60, like 50–60 years.  [Dispatcher]: Okay, about 60 years old, got it.  Is she awake and talking to you right now, yes or no?  [Caller]: Yes, yes, she awake.  She talk, but… short words only. She… uh… hard to breathe, little.  [Dispatcher]: Okay, stay right with her.  Is the chest pain in the middle of her chest, and does it feel like pressure or something heavy on her chest?  [Caller]: Yes, middle here—*center* chest.  She say very heavy, like… uh… squeeze, like something sit on chest. And go a little to arm and jaw.  [Dispatcher]: Okay, that sounds serious, help is coming fast.  Is she allergic to any medicine that you know of, yes or no?  [Caller]: Uh… I, I no know, sorry.  She my friend, but I no know allergy, no.  [Dispatcher]: Okay, no problem.  Is she still breathing on her own right now—yes or no?  [Caller]: Yes, yes, she breathing.  But… uh… fast, little… and not so good.  [Caller]: Hello? Sorry, you say again? I no hear.  She still here, she breathe, but she say more pain now.  You tell me what do, please?  [Caller]: Hello? I… I no hear you, sorry.  She… uh… she more bad now. Very pale, sweat more.  Breath faster—*huh, huh*—like this.  Please, you talk loud, slow. What I do now?  [Dispatcher]: Okay, I’m here, I hear you.  First, I need to know: is she still awake right now, yes or no?  [Caller]: Yes, yes, she still awake.  But… uh… eyes kind of… half close. She talk little, very weak.  [Dispatcher]: Okay, good job staying with her.  I want you to have her sit down on the floor or lie on her back with her head a little up—tell me when she is in that position.  [Dispatcher]: Okay, stay calm with me.  Is she now on the floor or on a bed, lying or sitting—where is she right now?  [Dispatcher]: I can’t hear you, so I’ll talk you through—just do this now:  Put the phone on speaker, stay next to her, and tell me if she is still awake—yes or no.  [Dispatcher]: If you can hear me, do this now: put your hand on her chest and tell me if her chest is moving up and down when she breathes—yes or no.  [Dispatcher]: If you can hear me, check her: is she awake—can she open her eyes or talk—yes or no?  [Caller]: Uh—yes, yes, she… she still awake.  Eyes open little, but she very weak. She say, “hurts… chest,” very soft.  Her chest still go up–down, but fast.  [Dispatcher]: Okay, good, keep talking to me.  Have her sit on the floor with her back against a wall or sofa, knees bent, and tell me when she is like that.  [Caller]: Uh—sorry, phone, I try move her.  Okay, she… she sit now, on floor, back on sofa, knees like this, bent.  She breathing, but still fast.  [Dispatcher]: Good, that’s perfect, keep her just like that.  Tell me, does she have any medicine for the heart or chest pain with her, like nitro pills or heart spray—yes or no?  [Caller]: Uh… she say before, she have… uh… nitro… little pill, long time.  But now she say, “I don’t know where is,” cannot find. So… no, we no have it here now.  [Dispatcher]: Okay, no problem, don’t look for it now, just stay with her.  Does she have diabetes, or does she use insulin or sugar medicine that you know—yes or no?  [Caller]: Yes, yes, she say she have sugar… uh, diabetes.  She take… uh… sugar medicine, pill, I think. Not… not needle, I don’t see needle.  [Dispatcher]: Okay, thank you, that helps.  Listen carefully: do you have any aspirin there, like baby aspirin or normal aspirin tablets—yes or no? | | |

# Supplementary Figures


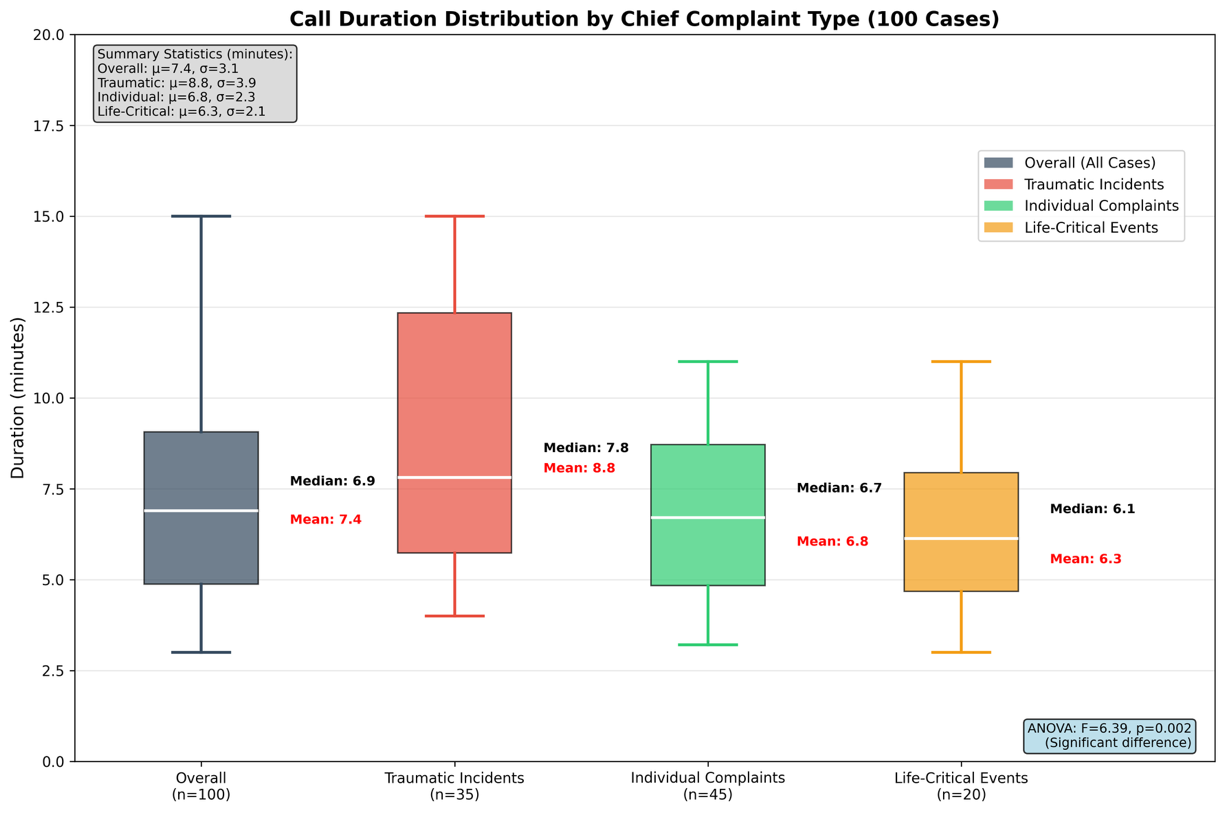


**Supplementary Figure. 1.** **Call duration distribution by chief complaint type (n = 100)**

A series of boxplots illustrates the distribution of simulated call durations across 100 cases, categorized by chief complaint type. The comparison includes four groups: Overall (n=100), Traumatic Incidents (n=35), Individual Complaints (n=45), and Life-Critical Events (n=20). For each category, the median (black labels) and mean (red labels) durations are annotated, revealing that Life-Critical Events had the shortest median duration (6.1 min) while Traumatic Incidents had the longest mean duration (8.8 min). A one-way ANOVA confirmed a statistically significant difference in duration among the groups (F = 6.39, p = 0.002), suggesting the system adapts its conversational timing based on scenario urgency. Duration Calculation: Call duration is a simulated metric, computed by summing the per-utterance time based on a standard human speaking rate. The calculation covers the entire six-phase call flow for both caller and dispatcher agents.


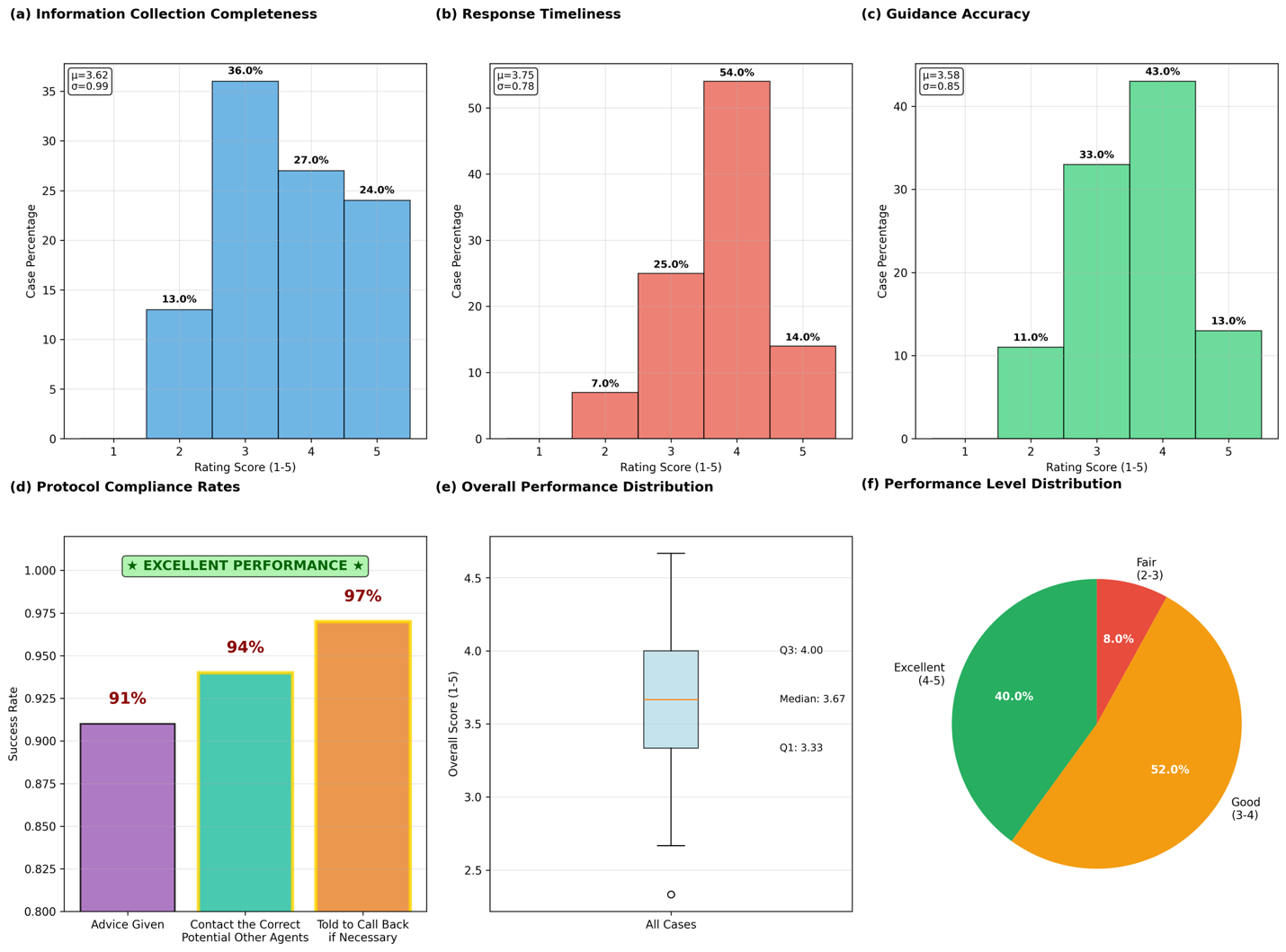
**Supplementary Figure. 2.** **Quantitative Evaluation of System Operational Performance**

A multifaceted, objective assessment of the DispatchMAS system's performance across 100 simulated cases is presented, with all metrics computed directly from system logs. The detailed scoring methodology for all metrics is provided in Supplementary Table S10. The analysis reveals a peak score of 3 (36.0% of cases) for information completeness (a), indicating competent data collection. The system demonstrated strong timeliness (b), with a majority of cases (54.0%) receiving a high score of 4, and robust guidance accuracy (c), with 43.0% of cases achieving a score of 4. As shown for key procedural steps (d), the system achieved high compliance rates for providing advice (91%), contacting correct external agents (94%), and instructing to call back if necessary (97%). Notably, the data in panel (d) were obtained through human expert assessment. The distribution of the composite Overall Performance Score (e) shows a median of 3.67. When categorized into performance tiers (f), a significant majority of cases were rated as "Good" (52.0%) or "Excellent" (40.0%), confirming the system's overall robust performance.

**
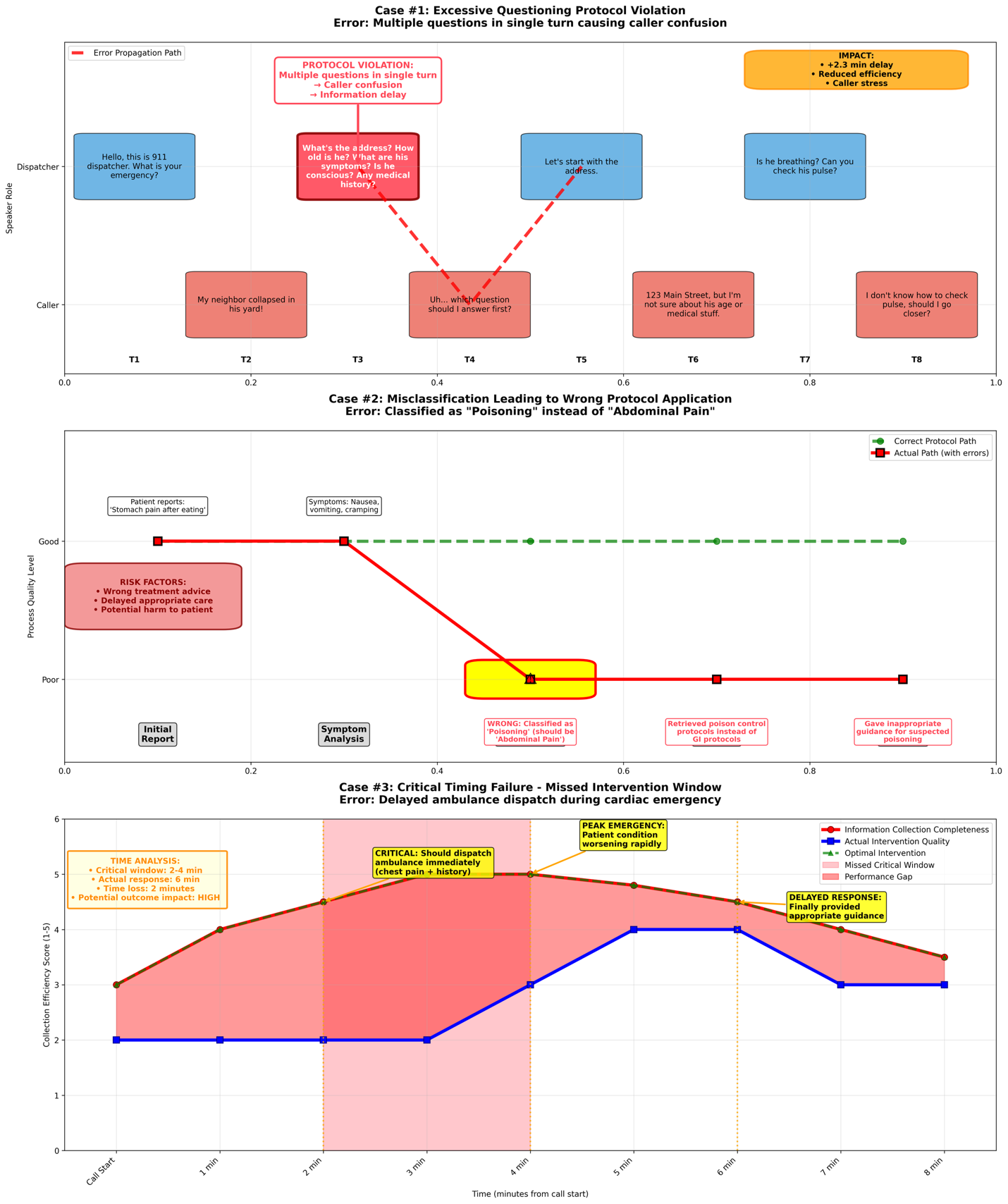
Supplementary Figure. 3.** **Analysis of representative system failure cases.**

This figure presents a qualitative analysis of three distinct failure modes to identify specific error patterns and their cascading effects . Case #1 illustrates a protocol violation where the dispatcher asks multiple questions in a single turn (T3), causing cognitive overload for the caller and leading to delays and reduced communication efficiency . Case #2 demonstrates a critical misclassification error where an initial report of "Abdominal Pain" is incorrectly classified as "Poisoning." This single error triggers a cascade of failures, causing the system to retrieve the wrong protocol and provide inappropriate guidance (actual path shown in red) . Case #3 depicts a critical timing failure during a simulated cardiac emergency. The system's actual intervention quality (blue line) lags significantly behind the ideal information collection completeness (red line), creating a large "Performance Gap" and missing the critical 2-4 minute intervention window for dispatching an ambulance .
